# Network analysis of sleep disorders, anxiety, and loneliness among the community-dwelling older adults

**DOI:** 10.3389/fpubh.2026.1827779

**Published:** 2026-06-10

**Authors:** Xiaonan Li, Lin Mao

**Affiliations:** 1 School of Sociology, Guizhou Minzu University, Guiyang, China; 2 School of Management, North Sichuan Medical College, Nanchong, China

**Keywords:** anxiety, community-dwelling older adults, loneliness, network analysis, sleep disorders, symptom

## Abstract

**Objective:**

To explore the relationships among sleep disorders, loneliness, and anxiety in community-dwelling older adults, and to provide empirical evidence for developing targeted intervention strategies to improve their overall health.

**Methods:**

A total of 1,637 community-dwelling older adults were recruited from Nanchong through convenience sampling. Sleep disorders, anxiety, and loneliness were assessed using the Pittsburgh Sleep Quality Index (PSQI), the Generalized Anxiety Disorder 7-item Scale (GAD-7), and the short form of the UCLA Loneliness Scale (ULS-8), respectively. Network analysis was conducted using R 4.5.1 to examine the symptom-level associations among sleep disorders, anxiety, and loneliness.

**Results:**

Most symptoms of sleep disorders, anxiety, and loneliness were positively correlated, forming a highly interconnected Gaussian graphical model (GGM) comorbidity network. In the GGM network, PSQI4, GAD2, and PSQI2 were identified as core symptoms, while PSQI4, ULS5, and PSQI2 were identified as bridge symptoms. Bayesian network analysis showed that ULS3 was the upstream node in the directed acyclic graph and pointed to 10 nodes, while GAD1 did not point to any nodes. Network comparison analysis further revealed no significant gender differences in the comorbidity network among older adults.

**Conclusion:**

This study provides a deeper understanding of the symptom-level relationships among sleep disorders, loneliness, and anxiety in community-dwelling older adults. The core and bridge symptoms identified in the GGM network may serve as key targets for the prevention and treatment of this comorbidity. In addition, the upstream node identified in the Bayesian network may provide useful insights for future longitudinal research and the development of interventions aimed at improving the overall health of community-dwelling older adults.

## 1 Introduction

Population aging is a pervasive and irreversible global trend that poses substantial challenges to social stability and well-being. The World Health Organization (WHO) predicts that the global population aged 60 years and older will reach 2.1 billion by 2050, accounting for approximately 22% of the total global population (1). As a country with an enormous population, China has already entered the stage of population aging (2). Recent data show that as of 2025, the number of people aged 60 years and older in China has reached 323 million, accounting for approximately 23% of the country’s total population (3). Although increased longevity is typically viewed as a positive outcome of public health advancements, ongoing population aging is accompanied by a range of complex psychological and behavioral health problems. Among these, sleep disorders, loneliness, and anxiety are especially prominent among older adults. Anxiety is a common mental disorder that often coexists with various medical illnesses in older adults (4). Loneliness is also prevalent in this population, with a higher prevalence in rural areas than in urban areas (5). Sleep disorders are another common health issue among older adults. A meta-analysis of more than 70,000 community-dwelling older adults from 13 countries found that 45% of participants experienced sleep disorders, with the prevalence increasing to 48% among those aged 70 years and older (6).

A large body of evidence indicates that anxiety and loneliness are closely associated and often co-occur among older adults (7–9). Specifically, anxiety may make older adults more vulnerable to feelings of loneliness (10), while persistent loneliness may further increase their risk of anxiety (11). Moreover, anxiety and loneliness are closely related at the symptom level, such that the presence of one symptom may increase the likelihood of the other (12, 13). These symptom-level interactions may contribute to the co-occurrence of anxiety and loneliness in older adults. For instance, one study found that 36.49% of frail older adults experienced comorbid anxiety, depression, and loneliness (9). This pattern of comorbidity often involves more complex clinical manifestations and poorer treatment outcomes (14).

Sleep disorders are also associated with both anxiety and loneliness (15, 16). Empirical evidence has shown that most anxiety disorders are significantly associated with poorer sleep quality. Among these disorders, social phobia and generalized anxiety disorder have the strongest associations with the Pittsburgh Sleep Quality Index (17). Further research has reported a bidirectional association between anxiety and sleep disorders: sleep disorders may increase the risk of anxiety by 1.9 times, while anxiety may raise the risk of sleep disorders by 1.2 times (18). Specifically, anxiety may contribute to sleep disturbances such as prolonged sleep latency and early morning awakening (19). These disturbances may, in turn, impair emotion regulation and further exacerbate anxiety symptoms, creating a vicious cycle (20). Furthermore, there is a bidirectional relationship between sleep disorders and loneliness. Specifically, loneliness may increase the risk of sleep disorders, as lonely older adults tend to have poorer sleep quality than their non-lonely counterparts (21). Conversely, insufficient sleep may contribute to social isolation and increased loneliness (22). Taken together, these findings suggest that anxiety, loneliness, and sleep disorders are closely interrelated among older adults. However, research on the symptom-level interactions among anxiety, loneliness, and sleep disorders remains insufficient, which limits both a systematic understanding of this comorbidity and the development of targeted interventions.

Although existing studies have demonstrated close associations among sleep disorders, anxiety, and loneliness, these conditions have generally been treated as single constructs or latent variables. Consequently, such research can identify overall associations among these variables but cannot clarify symptom-level interactions. In fact, certain symptoms within mental disorders may interact with and reinforce one another, thereby contributing to the development and maintenance of various mental disorders (23, 24). Network analysis is an emerging approach in psychological research for examining symptom-level interactions and addressing the limitations of traditional variable-centered methods. Based on graph theory, this approach treats symptoms as “nodes” and the partial correlations between symptoms as “edges” (25). Within such networks, core symptoms are those that are highly connected to other symptoms, whereas bridge symptoms are those that link different symptom communities. By identifying core and bridge symptoms, network analysis can provide valuable insights for developing targeted intervention strategies (26).

Network analysis has been increasingly applied to investigate symptom-level relationships in mental disorders, such as anxiety and depression (27, 28). However, most previous studies have relied on the Gaussian graphical model (GGM), which can reveal associations among symptoms but cannot infer their directional relationships (29). Bayesian networks offer an alternative network estimation approach for exploring potential probabilistic directional associations among symptoms based on cross-sectional data (29). For example, a study found that the symptom “obinterfer” was located at the top of the Bayesian network of obsessive-compulsive disorder (OCD) and depression. Accordingly, this symptom was considered an upstream symptom in the directed network and a potential priority target for intervention (30). However, Bayesian networks estimated from cross-sectional data can only reveal probabilistic directional associations among symptoms and cannot provide definitive evidence of causal relationships. The resulting findings should therefore be cautiously interpreted.

In this study, we used network analysis to examine the symptom-level relationships among sleep disorders, anxiety, and loneliness in community-dwelling older adults. Specifically, we first constructed an undirected symptom network using a Gaussian graphical model, identified core and bridge symptoms, and evaluated the accuracy and stability of the network. Second, we constructed a flow network to visualize the associations between core symptoms and other symptoms. Third, we employed Bayesian network estimation to construct directed acyclic graphs based on cross-sectional data, thereby exploring potential probabilistic directional associations among symptoms. Finally, we compared the GGM networks of older men and women using visualization and statistical tests to examine gender differences in the comorbidity network.

## 2 Methods

### 2.1 Participants

A convenience sampling method was used to recruit community-dwelling older adults in Nanchong City, China. The eligibility criteria for participants were as follows: (1) living in Nanchong City; (2) aged 60 years or older; and (3) being able to independently understand and complete the three scales: the Pittsburgh Sleep Quality Index (PSQI), the Generalized Anxiety Disorder 7-item Scale (GAD-7), and the short form of the UCLA Loneliness Scale (ULS-8). A total of 1,752 questionnaires were collected. After excluding invalid questionnaires, 1,637 valid questionnaires were retained for analysis, yielding a valid response rate of 93.44%. Table 1 presents the sociodemographic characteristics of the participants. The mean age of the sample was 73.27 years (SD = 5.88). Among the participants, 975 were female (59.56%) and 662 were male (40.44%). Most participants had an educational level of elementary school or below (76.12%), lived in rural areas (65.97%), and were married or cohabiting (75.14%). The research protocol was reviewed and approved by the Ethics Review Committee of the School of Sociology, Guizhou Minzu University. The study was conducted in accordance with relevant ethical regulations. Before data collection, all participants were informed of the purpose, procedures, and confidentiality measures of the study. Participants’ personal information was kept confidential, and all collected data were used only for academic research purposes.

**Table 1:** Sociodemographic characteristics of the sample (*N* = 1,637).

Variable	*N* (%)/Mean ± SD
Age (years)	73.27 ± 5.88
Gender	
Female	975 (59.56%)
Male	662 (40.44%)
Education level	
Illiterate	531 (32.44%)
Primary school	715 (43.68%)
Junior high school or above	391 (23.89%)
Residence	
Rural	1,080 (65.97%)
Urban	557 (34.03%)
Marital status	
Married/cohabiting	1,230 (75.14%)
Other (widowed, divorced, separated, or never married)	407 (24.86%)

### 2.2 Measures

#### 2.2.1 Pittsburgh sleep quality index

Participants’ subjective sleep quality over the preceding month was assessed using the Pittsburgh Sleep Quality Index [PSQI; (31)]. The PSQI consists of 19 self-rated items and 5 other-rated items completed by a bedmate or roommate. The 19th self-rated item and the 5 other-rated items are not included in the scoring, and the remaining 18 self-rated items are categorized into 7 components: subjective sleep quality, sleep duration, habitual sleep efficiency, sleep latency, use of sleep medication, daytime dysfunction, and sleep disturbances. Each component is scored from 0 to 3, yielding a total PSQI score ranging from 0 to 21. Higher total scores indicate poorer sleep quality. The PSQI has been shown to be suitable for use among Chinese older adults, and its reliability and validity have been tested (32). In this study, the PSQI showed acceptable internal consistency, with a Cronbach’s alpha coefficient of 0.754.

#### 2.2.2 Generalized anxiety disorder 7-item scale

Participants’ generalized anxiety symptoms over the past 2 weeks were assessed using the Generalized Anxiety Disorder 7-item Scale [GAD-7; (33)]. The GAD-7 consists of 7 items: feeling nervous, uncontrollable worry, excessive worry, trouble relaxing, restlessness, irritability, and feeling afraid. Each item is rated on a 4-point Likert scale according to the frequency of anxiety symptoms (0 = not at all; 1 = several days; 2 = more than half the days; 3 = nearly every day). The total score ranges from 0 to 21, with higher scores indicating more severe anxiety. The GAD-7 has been shown to be suitable for use among Chinese older adults (34). In the present study, the scale also showed excellent internal consistency, with a Cronbach’s alpha coefficient of 0.886.

#### 2.2.3 The short form of the UCLA loneliness scale

The UCLA Loneliness Scale (ULS) is a self-report instrument designed to assess loneliness (35) and has been widely applied in empirical studies. However, the original scale contains 20 items, which may increase participants’ response burden. Therefore, the present study adopted the short version of the scale, namely the 8-item UCLA Loneliness Scale [ULS-8; (36)]. The ULS-8 consists of 8 items, including 6 negatively worded items and 2 positively worded items. Participants rate each item on a 4-point Likert scale according to their feelings (1 = never; 2 = sometimes; 3 = often; 4 = always). In this study, the ULS-8 was used to assess loneliness among older adults, and the scores of the 2 positively worded items were reverse-coded. The total score ranges from 8 to 32, with higher scores indicating greater loneliness. The ULS-8 has been extensively used in empirical research on older adults and has demonstrated good reliability (37). In the present study, the ULS-8 also demonstrated acceptable internal consistency, with a Cronbach’s alpha coefficient of 0.767.

### 2.3 Statistical methods

#### 2.3.1 Estimation of GGM network

This study constructed a GGM network of anxiety, sleep disorders, and loneliness among community-dwelling older adults using the bootnet and qgraph packages in R version 4.5.1 (38). Given that all items were measured on ordinal Likert scales, Spearman’s rank-order correlation matrix was used as input for the network estimation, thereby avoiding the assumption of multivariate normality. The network was estimated using the estimateNetwork function, which employs the EBICglasso method by default. EBICglasso combines the Extended Bayesian Information Criterion (EBIC) and the Least Absolute Shrinkage and Selection Operator (LASSO) to obtain an optimally sparse estimate of the partial correlation matrix. Specifically, LASSO is a regularization technique that shrinks weak correlations to zero, thereby reducing false-positive edges. Meanwhile, EBIC balances model fit and model complexity by tuning the hyperparameter *γ* (39). In the GGM network, each node represents a symptom, and each edge represents the partial correlation between two nodes. A solid blue line denotes a positive correlation, whereas a dashed red line denotes a negative correlation. Edge thickness represents the strength of the association between nodes. Furthermore, node predictability was evaluated using the mgm package (40). Node predictability refers to the extent to which a given node can be explained by all other nodes in the GGM network. This metric was visualized as a ring surrounding each node, with a larger ring area indicating higher node predictability.

Node centrality was used to measure the relative importance of each node in the GGM network. Common centrality indicators include strength, betweenness, closeness, and expected influence (EI). In the present study, EI was used to assess node centrality because closeness and betweenness have shown relatively low stability (41, 42), whereas strength centrality does not account for the influence of negative edges. EI is calculated as the algebraic sum of the edge weights connected to a given node, with positive and negative edges retained in the calculation. A higher EI value indicates that a node has stronger overall connectivity and greater influence within the network (43). Nodes with high centrality are considered core symptoms and may serve as potential intervention targets for preventing mental disorders (25). Bridge symptoms are critical nodes that connect different symptom communities. Following the recommendations of Jones et al. (44), the present study used bridge expected influence (BEI) to identify bridge symptoms in the GGM network. BEI measures the total weight of edges linking a node to nodes in other symptom communities. EI and BEI were estimated using the qgraph and networktools packages in R. According to network psychiatry theory, bridge nodes play an important role in the onset and maintenance of comorbidity (45). Therefore, interventions targeting potential bridge symptoms may help prevent the development of comorbidity.

The study also systematically evaluated the accuracy and stability of the GGM network to assess the robustness and reliability of the network analysis. First, the 95% confidence intervals (CIs) of edges were estimated using a non-parametric bootstrap with 1,500 resamples using the bootnet package to assess the precision of edge weight estimates. The width of the CIs reflects the estimation precision of edge weights: narrower CIs indicate higher precision, whereas wider CIs indicate greater uncertainty. Moreover, a case-dropping bootstrap analysis was conducted to assess the stability of node centrality. This approach randomly removes portions of the sample and then re-estimates the GGM network to examine whether the ranking of node centrality indices remains stable. To quantify this stability, the corStability function in the bootnet package was used to calculate the correlation stability (CS) coefficient. A CS coefficient greater than 0.5 indicates relatively high stability of node centrality. Finally, bootstrap difference tests were conducted to evaluate whether edge weights and centrality indices differed significantly within the GGM network (*α* = 0.05).

#### 2.3.2 Estimation of the flow network

A flow network is a visualization method used to display the hierarchical connectivity patterns between a node of interest and other nodes in the network. In this method, the node of interest, typically the core symptom, is positioned on the left, while the remaining nodes are arranged vertically on the right in descending order according to the strength of their associations with that node. This layout can effectively distinguish nodes that are directly or indirectly connected to the core symptom. Based on the GGM network of anxiety, sleep disorders, and loneliness among community-dwelling older adults, the present study used the flow function from the qgraph package in R 4.5.1 to generate a flow network diagram, which clearly visualizes the strength of the associations between each node and the core node.

#### 2.3.3 Estimation of Bayesian networks

A Bayesian network is a network estimation method that can be used to explore potential directional associations among variables. In this study, the Bayesian hill-climbing algorithm implemented in the bnlearn package was used to estimate a directed network to examine the potential directional associations among sleep disorders, anxiety, and loneliness. The hill-climbing algorithm iteratively optimizes the network structure by adding, deleting, or reversing edges to identify the model with the lowest Bayesian Information Criterion (BIC), thereby determining the optimal network structure (46). To improve the robustness of the directed network structure, 2,000 bootstrap resamples were generated, and only edges with relatively high stability across the resampled datasets were retained. The directed acyclic graph (DAG) was then constructed to visualize the Bayesian network and identify potential directional associations among symptoms. In the DAG, edge thickness represents the relative importance of the directed association: edges that appear more frequently across bootstrap samples or contribute more to model fitting are displayed as thicker lines. Additionally, nodes positioned higher in the layout may be regarded as upstream nodes, suggesting their relatively greater importance within the directed network structure (30).

#### 2.3.4 Network comparison

This study systematically compared GGM networks between male and female participants to examine gender differences in the comorbidity network of sleep disorders, anxiety, and loneliness among older adults. First, the network comparison test (NCT) was performed using the NetworkComparisonTest package with 2,000 permutations to examine differences in global strength, network structure, edge weights, and node centrality between the two groups. A *p*-value < 0.05 was considered statistically significant (47). Additionally, community detection algorithms were applied to compare the differences in community structures between male and female networks. Specifically, the spin glass algorithm implemented in the igraph package was employed to identify potential subnetworks in the comorbidity networks. This method is a modularity-based subnetwork detection approach, which assumes that connections between nodes within a subnetwork are stronger than connections between nodes from different subnetworks (48).

## 3 Results

### 3.1 Descriptive statistics

**Figure 1:**
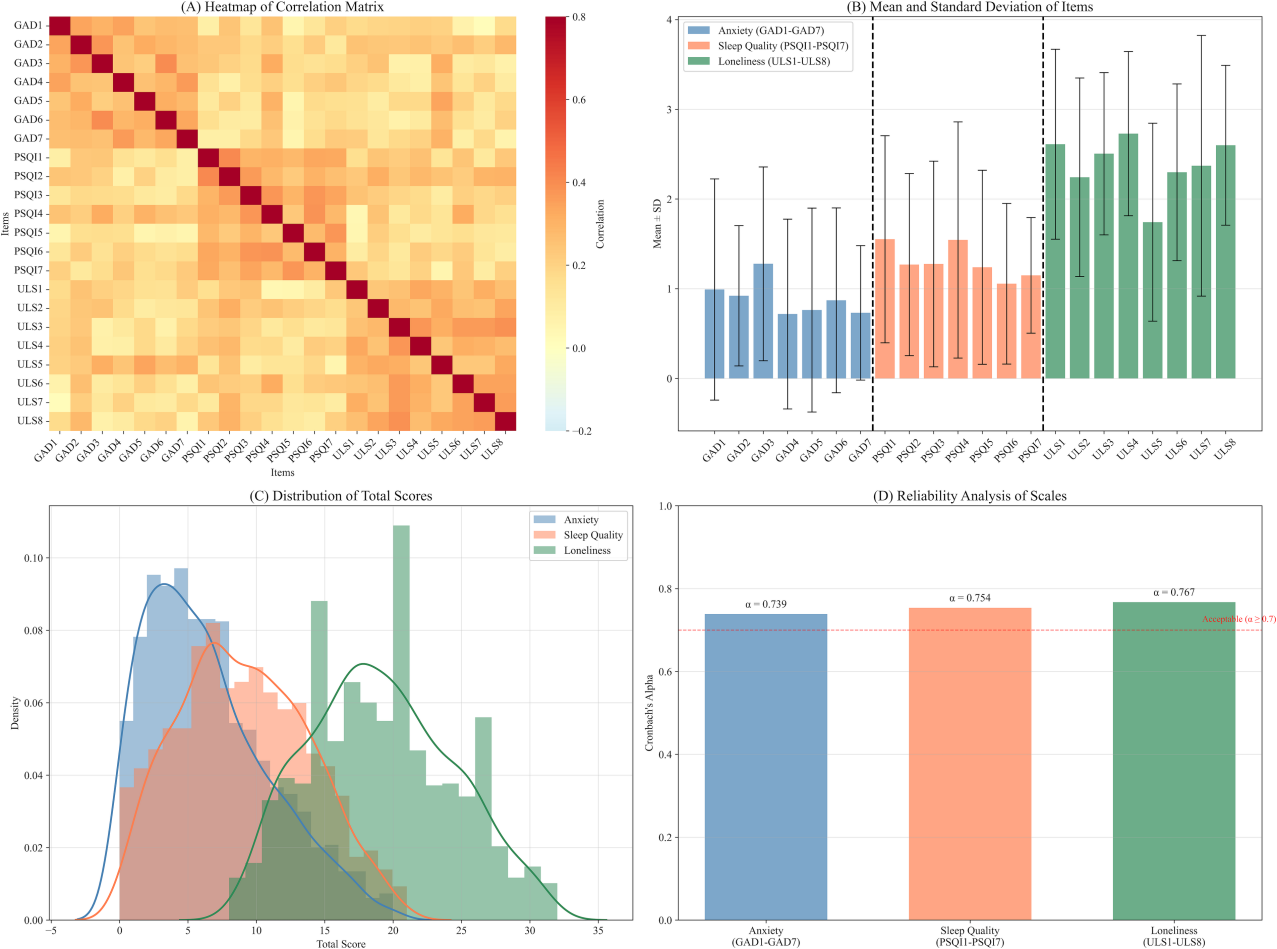
Descriptive statistics and reliability analyses of the three scales. **(A)** Correlation matrix of all items. **(B)** Mean scores and standard deviations for each item. **(C)** Histograms of total scores for the three scales with fitted normal distribution curves. **(D)** Cronbach’s α coefficients indicating the internal consistency reliability of the three scales.

Table 2 presents the means and standard deviations of all items across the three scales. The mean total scores for the GAD-7, PSQI, and ULS-8 were 6.27 (SD = 4.47), 9.08 (SD = 4.69), and 19.10 (SD = 5.26), respectively. The items with the highest mean scores on the GAD-7, PSQI, and ULS-8 were GAD3 (*M* = 1.28, SD = 1.08), PSQI1 (*M* = 1.55, SD = 1.15), and ULS4 (*M* = 2.73, SD = 0.91), respectively. Figure 1 presents the descriptive statistics and reliability analyses for the three scales. Specifically, Figure 1A shows a correlation matrix heatmap of all items. The results indicated that most items of sleep disorders, anxiety, and loneliness were positively correlated, with darker colors representing stronger correlations. This suggested that these items were interrelated to varying degrees, thereby providing a feasible basis for subsequent network analysis. Figure 1B shows the means and standard deviations for each item, visually presenting the central tendency and dispersion of different symptoms. Figure 1C shows histograms with fitted density curves, indicating that the total scores of the three scales were approximately normally distributed. Figure 1D presents the internal consistency results of the three scales. The Cronbach’s *α* coefficients for all scales exceeded the acceptable threshold of 0.70, demonstrating high internal consistency among items and confirming the reliability of the measurements.

**Table 2:** Descriptive statistics and predictability of items.

Item	Item description	Abbreviation	Mean (SD)	Predictability
GAD	Generalized anxiety disorder	–	6.27 (4.47)	–
GAD1	1. Feeling nervous, anxious, or on edge	Feeling nervous	0.99 (1.23)	0.345
GAD2	2. Not being able to stop or control worrying	Uncontrollable worry	0.92 (0.78)	0.346
GAD3	3. Worrying too much about different things	Excessive worry	1.28 (1.08)	0.379
GAD4	4. Trouble relaxing	Trouble relaxing	0.72 (1.06)	0.306
GAD5	5. Being so restless that it is hard to sit still	Restlessness	0.76 (1.14)	0.286
GAD6	6. Becoming easily annoyed or irritable	Irritability	0.87 (1.03)	0.304
GAD7	7. Feeling afraid as if something awful might happen	Feeling afraid	0.73 (0.75)	0.316
PSQI	Pittsburgh sleep quality index	–	9.08 (4.69)	–
PSQI1	1. Subjective sleep quality	Subjective sleep quality	1.55 (1.15)	0.340
PSQI2	2. Sleep latency	Sleep latency	1.27 (1.01)	0.380
PSQI3	3. Sleep duration	Sleep duration	1.28 (1.15)	0.334
PSQI4	4. Habitual sleep efficiency	Habitual sleep efficiency	1.54 (1.32)	0.402
PSQI5	5. Sleep disturbances	Sleep disturbances	1.24 (1.08)	0.255
PSQI6	6. Use of sleeping medication	Use of sleeping medication	1.06 (0.89)	0.311
PSQI7	7. Daytime dysfunction	Daytime dysfunction	1.15 (0.64)	0.359
ULS	The UCLA loneliness scale	–	19.10 (5.26)	–
ULS1	1. I lack companionship	Lack companionship	2.61 (1.06)	0.303
ULS2	2. There is no one I can turn to	No one to turn to	2.24 (1.11)	0.278
ULS3	3. I am an outgoing person	Outgoing person	2.51 (0.90)	0.348
ULS4	4. I feel left out	Feel left out	2.73 (0.91)	0.302
ULS5	5. I feel isolated from others	Feel isolated	1.74 (1.10)	0.391
ULS6	6. I can find companionship when I want it	Find companionship	2.30 (0.98)	0.327
ULS7	7. I am unhappy being so withdrawn	Unhappy being withdrawn	2.37 (1.45)	0.348
ULS8	8. People are around me but not with me	Present but disconnected	2.60 (0.89)	0.374

### 3.2 Analysis results of the GGM network

**Figure 2:**
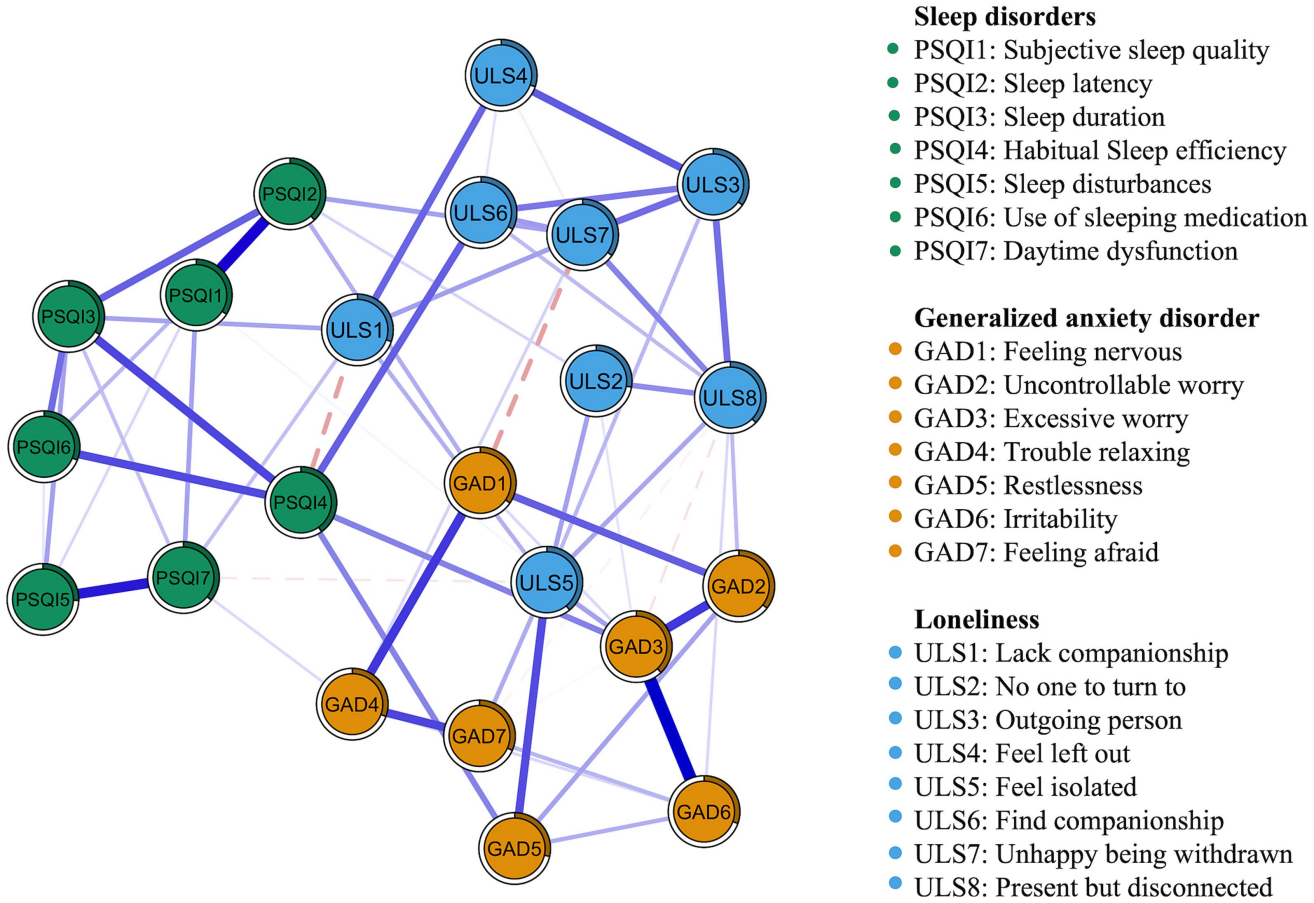
GGM network of sleep disorders, anxiety, and loneliness symptoms.

**Figure 3:**
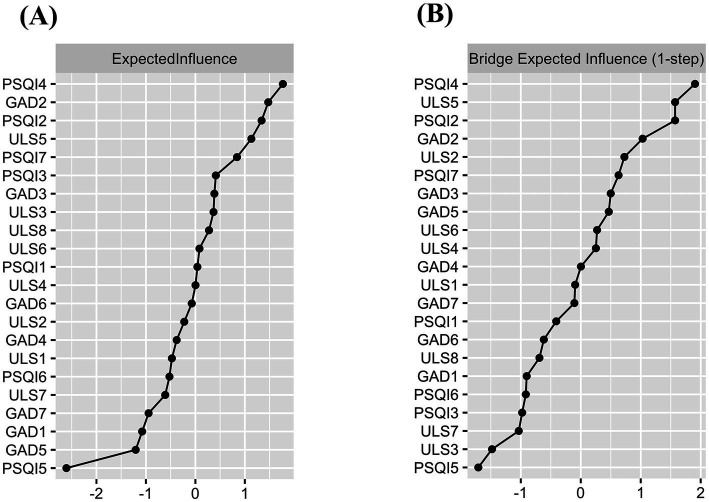
Expected influence and bridge expected influence of symptoms. **(A)** Ranking of EI for each node in the GGM network. **(B)** Ranking of BEI for each node in the GGM network.

Figure 2 presents the GGM network of sleep disorders, anxiety, and loneliness symptoms among community-dwelling older adults. Of the 231 possible edges, the GGM network had 176 non-zero edges, resulting in a network density of 0.762. The global network strength was 11.959, reflecting strong overall associations among symptoms of sleep disorders, anxiety, and loneliness. The three edges with the largest weights were GAD3–GAD6 (edge weight = 0.24), PSQI1–PSQI2 (edge weight = 0.22), and PSQI5–PSQI7 (edge weight = 0.21). Node predictability is represented as an outer ring around each node in Figure 2, indicating the proportion of variance in that node explained by its neighboring nodes. PSQI4 showed the highest predictability value of 0.402. Across all symptoms, the mean predictability was 0.333, suggesting that neighboring nodes explained an average of 33.3% of the variance for each symptom. Detailed predictability results are presented in Table 2. Figures 3A,B show the EI and BEI for each symptom, respectively. PSQI4, GAD2, and PSQI2 showed the highest standardized EI values, indicating that these symptoms had greater overall influence within the network. PSQI4, ULS5, and PSQI2 had the highest standardized BEI values, suggesting that these symptoms may serve as important bridge symptoms linking sleep disorders, anxiety, and loneliness.

**Figure 4:**
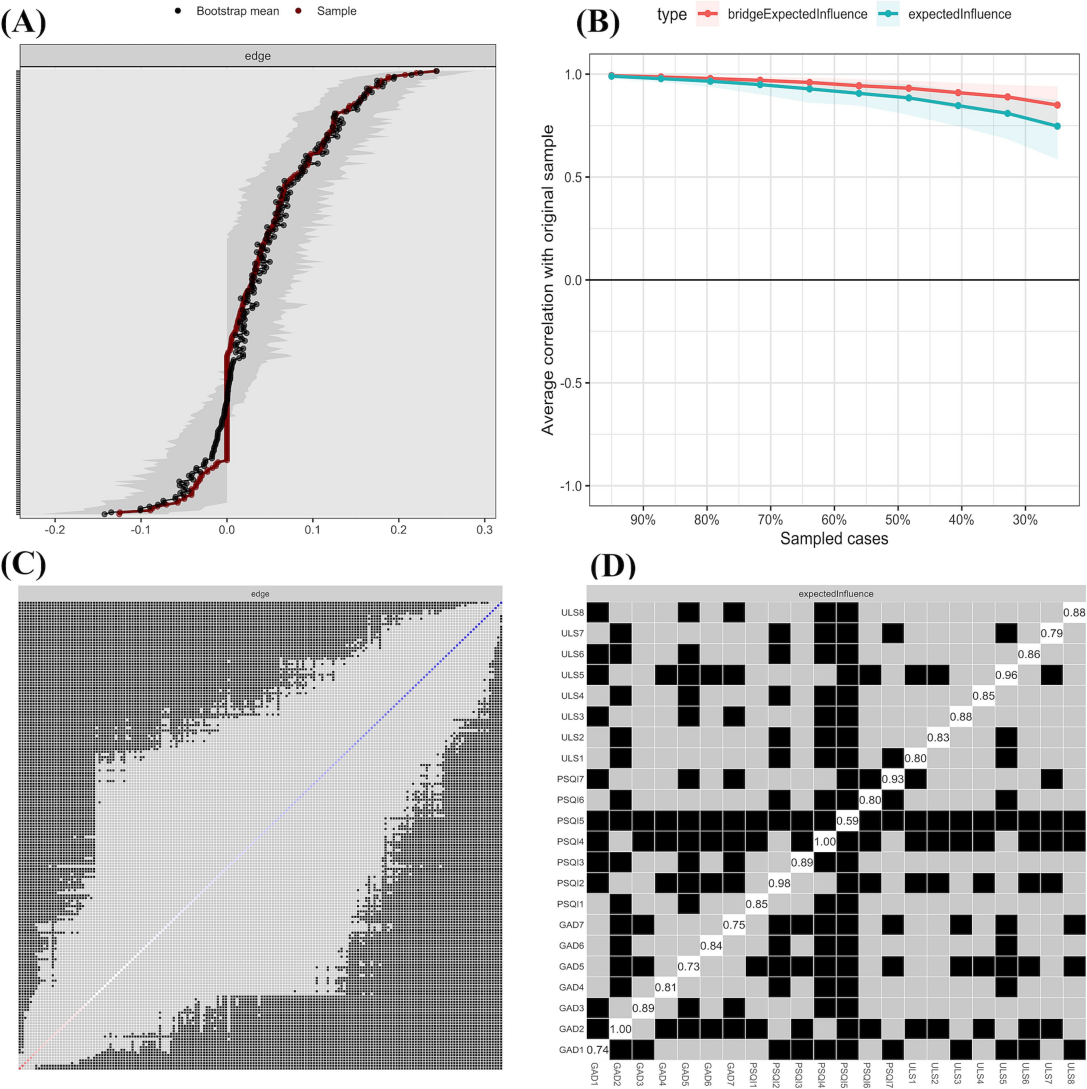
Accuracy and stability analyses of the GGM network. **(A)** The 95% confidence intervals for all non-zero edge weights. **(B)** The case-dropping bootstrap stability analysis results. **(C)** The results of the bootstrapped difference test for edge weights. **(D)** The results of the bootstrapped difference test for node centrality metrics.

Figure 4 shows the accuracy and stability analyses of the GGM network. Figure 4A presents the 95% confidence intervals for each edge weight, most of which are relatively narrow, indicating high accuracy of the edge estimates. Figure 4B presents the case-dropping bootstrap analysis results, which indicate that the centrality indices remained stable even when the sample size was reduced. Specifically, the CS coefficients of EI and BEI were 0.67 and 0.75, respectively, both exceeding the recommended threshold of 0.50 and confirming the reliability of these centrality metrics. Supplementary Table S1 provides the detailed results of the case-dropping bootstrap analysis. Figures 4C,D display the bootstrapped difference tests for edge weights and node centrality metrics, respectively. In these plots, black squares denote statistically significant differences, while gray squares indicate non-significant differences. In this study, most node centrality metrics and edge weights exhibited significant differences, supporting the structural stability of the GGM network.

### 3.3 Analysis results of the flow network

**Figure 5:**
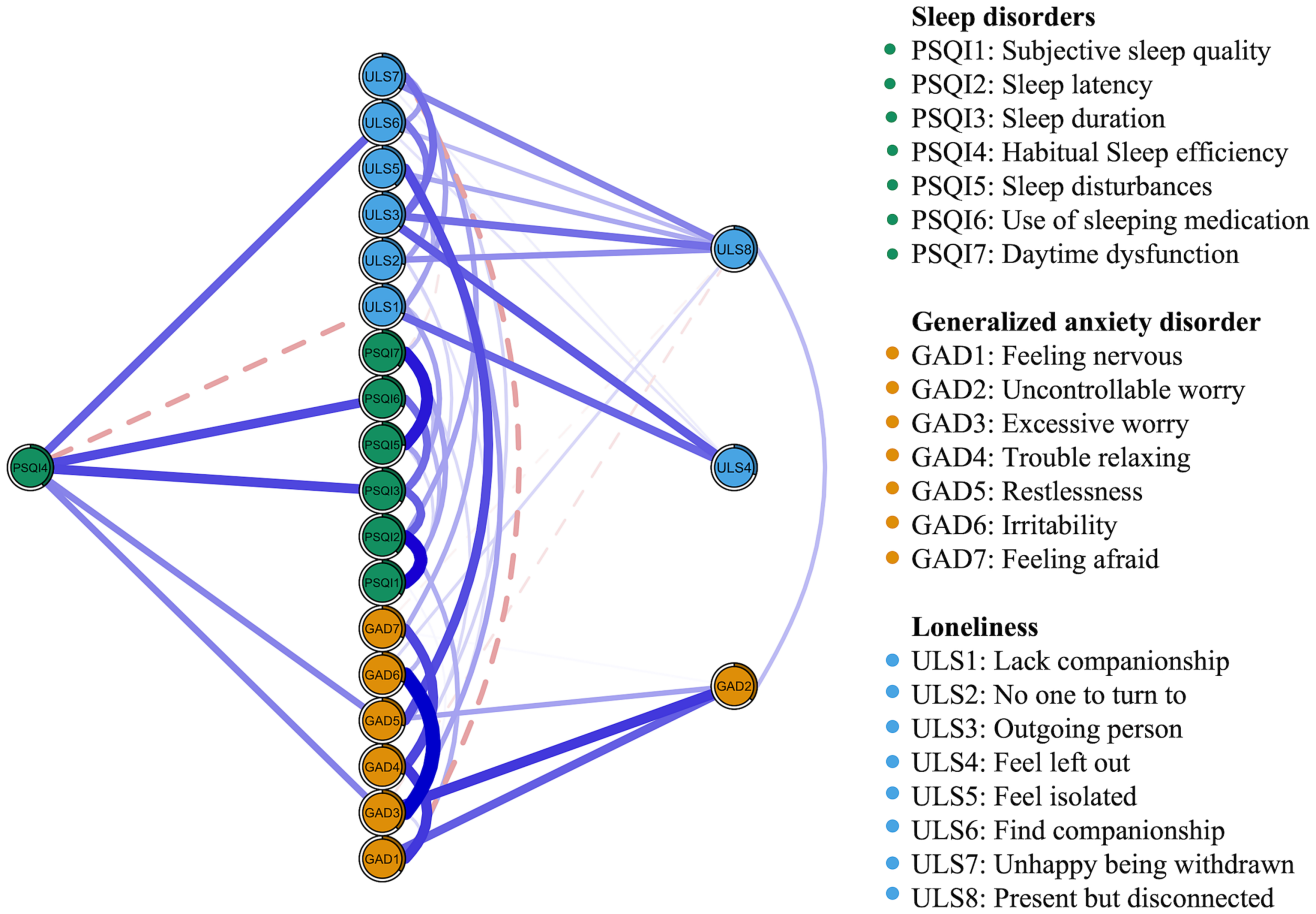
Flow network based on the GGM network.

Figure 5 depicts the flow network among sleep disorders, anxiety, and loneliness symptoms, illustrating the association patterns between the core node and other nodes in the comorbidity network. As PSQI4 was identified as the core symptom in the GGM network, it was selected as the starting node for the flow network analysis. The results revealed that 18 symptoms were directly connected to PSQI4, while 3 nodes were indirectly linked to it. Among the directly connected symptoms, PSQI3 (edge weight = 0.18), PSQI6 (edge weight = 0.18), and ULS6 (edge weight = 0.16) exhibited the strongest connections with PSQI4. These findings suggest that PSQI4 may play a central role in the comorbidity network by first influencing directly connected symptoms and subsequently affecting indirectly connected symptoms through these direct associations. Therefore, PSQI4 and its most strongly associated symptoms may represent priority targets for intervention, as interventions targeting these symptoms may help weaken their direct connections with other symptoms, thereby alleviating the mutual reinforcement among symptoms within the comorbidity network.

### 3.4 Analysis results of DAG network

**Figure 6:**
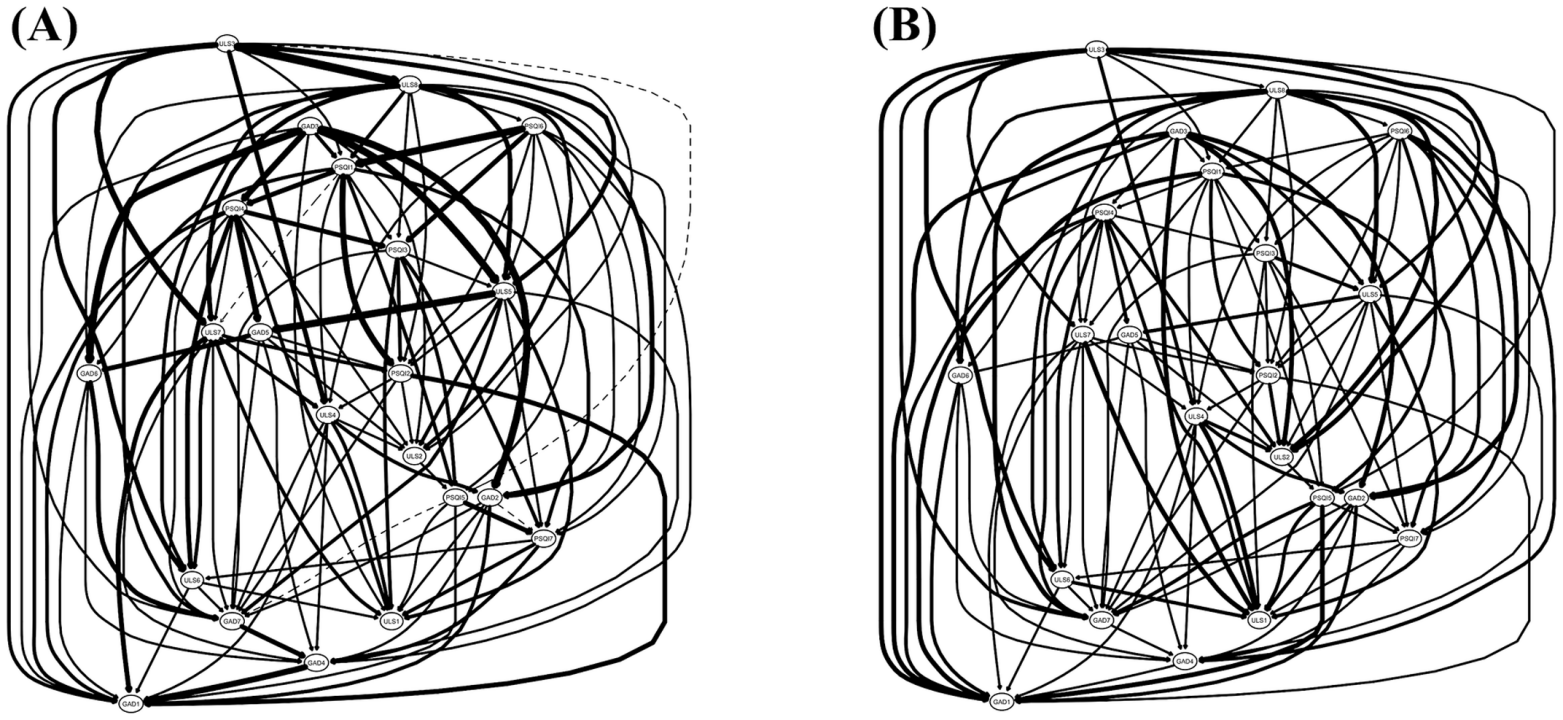
DAG networks for sleep disorders, anxiety, and loneliness symptoms. **(A)** The DAG based on Bayesian information criterion. **(B)** The DAG based on probability.

Figure 6A displays a directed acyclic graph (DAG) illustrating the directional associations among sleep disorders, anxiety, and loneliness symptoms. In this graph, arrow thickness reflects the magnitude of the BIC change when an arrow is removed from the DAG. Specifically, larger BIC changes indicate greater contributions to model fit and correspond to thicker arrows (30). The most influential arrows in the DAG network were ULS3 → ULS8 (BIC = −130.99), GAD3 → GAD2 (BIC = −106.06), and GAD3 → GAD6 (BIC = −93.01). As an upstream node, ULS3 directly pointed to 10 nodes, whereas GAD1 was positioned at the bottom of the DAG and did not point to any nodes. In addition, Figure 6B displays the probability-based DAG. In this figure, arrow thickness reflects the probability that a given edge appears across bootstrap networks, with higher occurrence probabilities represented by thicker arrows. The most critical arrows in the DAG network were ULS3 → GAD1 (*p* = 81.62%), PSQI5 → GAD1 (*p* = 81.08%), and ULS3 → ULS2 (*p* = 80.95%). Consistent with the BIC-based DAG, ULS3 was located at the highest position of the DAG and pointed to 10 nodes, whereas node GAD1 was located at the bottom of the DAG and did not point to any nodes. Detailed results are provided in Supplementary Tables S2, S3. These findings suggest that ULS3 occupies a relatively upstream position in the DAG network and may exert directional influence on multiple symptoms. Therefore, ULS3 may be considered a potentially key symptom for future clinical attention and early intervention.

### 3.5 Analysis results of network comparison

**Figure 7:**
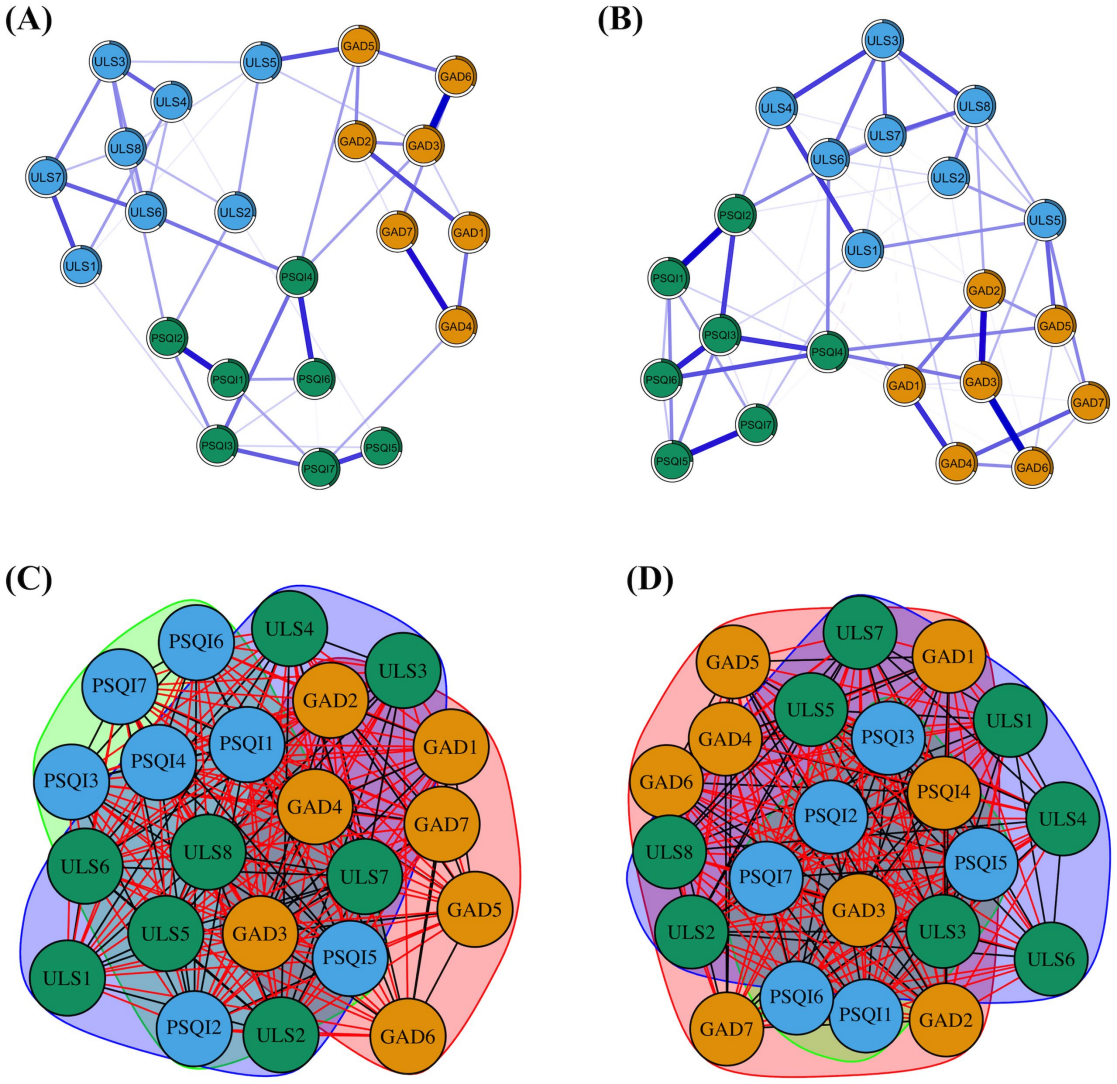
GGM networks and community structures across genders. **(A)** The GGM network of males. **(B)** The GGM network of females. **(C)** The community structure of males. **(D)** The community structure of females.

Figures 7A,B show the GGM comorbidity networks for older adults of different genders. The results of the network invariance test (*M* = 0.103, *p* = 0.810) and the global strength invariance test (*S* = 0.518, *p* = 0.485) indicated no significant overall differences in the GGM network between genders. Centrality invariance test also showed no significant gender differences in the centrality indicators of any node (*p* > 0.05). The edge invariance test identified only 8 edges with significant gender differences: PSQI3–PSQI7 (*p* = 0.028), GAD3–PSQI5 (*p* = 0.020), GAD4–PSQI4 (*p* = 0.048), GAD4–ULS4 (*p* = 0.048), ULS2–ULS4 (*p* = 0.033), ULS1–ULS7 (*p* = 0.028), GAD5–ULS1 (*p* = 0.026), and ULS2–ULS6 (*p* = 0.040). Detailed analysis results are shown in Supplementary Tables S4, S5. In addition, Figures 7C,D show the community detection results for the gender subgroups. The results showed that symptoms in both subgroups were divided into three communities, although the specific composition of these communities differed between the subgroups.

## 4 Discussion

In this study, PSQI4 (Habitual Sleep Efficiency), GAD2 (Uncontrollable Worry), and PSQI2 (Sleep Latency) were identified as core symptoms within the GGM network of sleep disorders, anxiety, and loneliness, reflecting their central role and significant influence within the comorbidity network. This finding is consistent with previous research. For example, a study on breast cancer patients identified “Habitual Sleep Efficiency” as a core symptom in the comorbidity network of sleep quality, depression, and anxiety (49). Similarly, research on individuals with insomnia reported that “Habitual Sleep Efficiency” exhibited high strength centrality in the comorbidity network of anxiety, depression, and sleep disturbances (50). The network analysis further identified PSQI4 (Habitual Sleep Efficiency), ULS5 (Feel Isolated), and PSQI2 (Sleep Latency) as bridge symptoms linking sleep disorders, anxiety, and loneliness symptoms in community-dwelling older adults. This finding is consistent with previous studies. For instance, research on patients with subthreshold depression demonstrated that “Habitual Sleep Efficiency” functioned as a key bridge symptom connecting insomnia, anxiety, and depression symptoms (51). Similarly, another study involving the older adults also highlighted “Habitual Sleep Efficiency” as a bridge symptom connecting emergent neuropsychiatric and sleep disorder symptoms in the subthreshold depression group (52).

It is worth noting that “Habitual Sleep Efficiency” emerges as the most critical core and bridge symptom in the GGM network. Habitual sleep efficiency, defined as the ratio of total sleep time to time spent in bed attempting to sleep, reflects both sleep quality and efficiency. One underlying mechanism may explain its dual role as both a core and bridge symptom. Specifically, poor sleep efficiency can exacerbate anxiety and loneliness by reducing emotional resilience and increasing stress reactivity (53, 54). In turn, elevated anxiety and perceived loneliness can further impair sleep efficiency, forming a feedback loop that reinforces the centrality and bridging function of PSQI4 within the comorbidity network (16, 55). Previous studies have highlighted that interventions targeting core and bridge symptoms can both alleviate surrounding symptoms and weaken the links between comorbid symptoms, thereby enhancing overall treatment efficacy (25, 26, 56). Therefore, intervening on the symptom of “Habitual Sleep Efficiency” is essential when managing the comorbidity of sleep disorders, anxiety, and loneliness in community-dwelling older adults. For example, employing cognitive behavioral therapy (CBT), limiting time spent in bed, and promoting regular physical activity (57, 58) may help improve sleep efficiency and enhance overall health in this population.

Bayesian network analysis revealed probabilistic directional relationships among symptoms of sleep disorders, anxiety, and loneliness. In the DAG, loneliness symptoms exhibited 17 directed connections to anxiety symptoms and 11 directed connections to sleep disorder symptoms, suggesting that they occupied an upstream position in the symptom network. Moreover, the loneliness node ULS3 (Outgoing Person) served as the initial node of the DAG network, potentially exerting directional influence on multiple symptoms. These results collectively suggest that loneliness might be an earlier and more impactful factor in the comorbidity among sleep disorders, anxiety, and loneliness. This finding is consistent with previous studies that have identified loneliness as a predictor of anxiety and sleep disorders. For example, a longitudinal mediation study of older adults in the United States reported that perceived loneliness predicted subsequent increases in anxiety (7). Another Swedish cohort study found that baseline loneliness significantly increased the risk of sleep disturbances at follow-up, with continuously lonely individuals facing a 92% higher risk than those who were not lonely (59). Therefore, it is essential to formulate and implement proactive intervention strategies targeting loneliness in order to alleviate the severity of comorbidity and promote the overall health of older adults. Notably, although ULS3 emerged as an initial node in the Bayesian network, this finding should be interpreted with caution. As a reverse-coded item, higher scores on ULS3 reflect lower extraversion or reduced social engagement rather than the subjective experience of loneliness itself, suggesting that this result may be affected by measurement characteristics and warrants further verification.

The inferences derived from Bayesian networks based on cross-sectional data should be interpreted with caution due to several limitations. First, Bayesian networks assume that all relevant latent variables are included. Omitting key variables—such as unmeasured symptoms—may lead to spurious causal effects, where mere correlations are misinterpreted as causal links (60). In this study, the network model included only sleep disorders, anxiety, and loneliness, whereas previous research has suggested that factors such as depression, social support, and cognitive function may also influence the relationships among these constructs. Consequently, some edges in the current network may reflect omitted-variable effects or spurious associations. Future research should include these clinically and socially relevant variables to construct a more comprehensive network model. Second, causal inference in Bayesian networks relies on the Markov assumption, which is difficult to satisfy in complex mental disorders (61). Finally, Bayesian networks using directed acyclic graphs identify only unidirectional relationships, while feedback loops frequently occur among mental health symptoms. Accordingly, the results of the Bayesian network in this study should be interpreted as exploratory directional associations rather than strong evidence for causal relationships.

The study found no significant gender differences in the network structure or clustering patterns of sleep disorders, anxiety, and loneliness symptoms. This finding is consistent with previous research. For example, one study reported no gender differences in the symptom network structure of depression, anxiety, and sleep disorders among older adults during the COVID-19 lockdowns (62). These results indicate that despite differences in social roles and psychological characteristics between genders, the associations among sleep disorders, anxiety, and loneliness symptoms remain largely consistent. In conclusion, this study not only confirms the existence of comorbidity among community-dwelling older adults but also offers a preliminary comparison of symptom interrelations from a gender perspective, thereby providing a reference for the development of targeted intervention strategies.

This study not only enriches the network theory of comorbidity among community-dwelling older adults but also holds important implications for psychological intervention in this population. Nevertheless, several limitations should be noted. First, this study relied on cross-sectional data, which can reveal associations among symptoms but cannot establish clear causality. Future research could employ longitudinal panel data to clarify the causal mechanisms underlying comorbidity. Second, all data were collected via self-report measures, which may be subject to recall bias and could potentially influence the results. Third, potential symptomatic overlap between anxiety and sleep disorders—such as difficulty falling asleep due to worry—may inflate the observed associations within the network model. Future studies should use professional clinical evaluations and physiological sleep monitoring to mitigate these reporting biases and reduce symptom overlap. Finally, this study recruited participants from a single region, Nanchong City, using convenience sampling, with a relatively high proportion of rural older adults. Regional cultural factors, local social structures, and rural–urban differences may shape the patterns of relationships among loneliness, sleep disorders, and anxiety. Therefore, the findings of this study should be generalized to broader older adult populations with caution. Future studies should recruit more diverse samples from multiple regions and use probability sampling methods to improve the representativeness and external validity of the findings.

## 5 Conclusion

This study systematically examined the symptom-level relationships among sleep disorders, anxiety, and loneliness in community-dwelling older adults. The findings revealed strong positive associations among these symptoms, forming a highly interconnected comorbidity network. The estimated network showed good accuracy and effectively reflected the overall pattern of symptom associations. Centrality analysis identified PSQI4 (Habitual Sleep Efficiency) as both a core symptom and a bridge symptom within the comorbidity network. Based on this finding, flow network analysis was conducted using PSQI4 (Habitual Sleep Efficiency) as the starting point to further elucidate the symptoms directly or indirectly connected to this core symptom. Bayesian network analysis indicated potential directional relationships among symptoms, and ULS3 (Outgoing Person) may serve as an upstream node influencing the development of other symptoms. In addition, network comparison analysis showed no significant gender differences in the comorbidity network structure or symptom clustering patterns among older adults.

## Data Availability

The raw data supporting the conclusions of this article will be made available by the authors, without undue reservation.

## References

[1] World Health Organization (2025). Ageing and Health Geneva: World Health Organization Available online at: https://www.who.int/news-room/fact-sheets/detail/ageing-and-health (Accessed February 15, 2026).

[2] Han Y He Y Lyu J Yu C Bian M Lee L. Aging in China: perspectives on public health. Glob Health J. (2020) 4:11–7. doi: 10.1016/j.glohj.2020.01.002

[3] National Bureau of Statistics of China (2026) The Total national Population in 2025 Reached 1.40489 Billion, with Continuous Promotion of high-Quality Population Development. Available online at: https://www.stats.gov.cn/xxgk/jd/sjjd2020/202601/t20260119_1962338.html (Accessed February 15, 2026).

[4] Wolitzky-Taylor KB Castriotta N Lenze EJ Stanley MA Craske MG. Anxiety disorders in older adults: a comprehensive review. Depress Anxiety. (2010) 27:190–211. doi: 10.1002/da.20653, 20099273

[5] Savikko N Routasalo P Tilvis RS Strandberg TE Pitkälä KH. Predictors and subjective causes of loneliness in an aged population. Arch Gerontol Geriatr. (2005) 41:223–33. doi: 10.1016/j.archger.2005.03.002, 15908025

[6] Fu T Guo R Wang H Yu S Wu Y. The prevalence and risk factors of sleep disturbances in community-dwelling older adults: a systematic review and meta-analysis. Sleep Breath. (2025) 29:110–4. doi: 10.1007/s11325-025-03267-6, 39982574

[7] Santini ZI Jose PE Cornwell EY Koyanagi A Nielsen L Hinrichsen C . Social disconnectedness, perceived isolation, and symptoms of depression and anxiety among older Americans (NSHAP): a longitudinal mediation analysis. Lancet Public Health. (2020) 5:e62–70. doi: 10.1016/S2468-2667(19)30230-0, 31910981

[8] Van Bogart K Scott SB Harrington KD Felt JM Sliwinski MJ Graham-Engeland JE. Examining the bidirectional nature of loneliness and anxiety among older adults in daily life. J Gerontol. (2023) 78:1676–85. doi: 10.1093/geronb/gbad105, 37527478 PMC10561887

[9] Wang X Zhu B Li J Li X Zhang L Wu Y . The moderating effect of frailty on the network of depression, anxiety, and loneliness in community-dwelling older adults. J Affect Disord. (2025) 375:508–16. doi: 10.1016/j.jad.2025.01.118 39862977

[10] Farhang M Álvarez-Aguado I Celis Correa J Toffoletto MC Rosello-Peñaloza M Miranda-Castillo C. Effects of anxiety, stress and perceived social support on depression and loneliness among older people during the COVID-19 pandemic: a cross-sectional path analysis. Inquiry. (2024) 61:00469580241273187. doi: 10.1177/00469580241273187, 39229739 PMC11375662

[11] Domènech-Abella J Mundó J Haro JM Rubio-Valera M. Anxiety, depression, loneliness and social network in the elderly: longitudinal associations from the Irish longitudinal study on ageing (TILDA). J Affect Disord. (2019) 246:82–8. doi: 10.1016/j.jad.2018.12.043, 30578950

[12] Dong B Li B Fan X Chen H Dang Z Li Z. A network analysis study of anxiety, depression and loneliness among middle-aged and elderly people in Xining area. BMC Psychol. (2025) 13:931. doi: 10.1186/s40359-025-03248-0, 40826485 PMC12359986

[13] Mi Y Ahn S Ren L. Exploring the interconnections of loneliness, anxiety, and depression among nursing students: a network analysis approach. Front Psych. (2025) 16:1537935. doi: 10.3389/fpsyt.2025.1537935, 40034186 PMC11873105

[14] Igbokwe CC Ejeh VJ Agbaje OS Umoke PIC Iweama CN Ozoemena EL. Prevalence of loneliness and association with depressive and anxiety symptoms among retirees in northcentral Nigeria: a cross-sectional study. BMC Geriatr. (2020) 20:153. doi: 10.1186/s12877-020-01561-4, 32326891 PMC7178938

[15] Alvaro PK Roberts RM Harris JK. A systematic review assessing bidirectionality between sleep disturbances, anxiety, and depression. Sleep. (2013) 36:1059–68. doi: 10.5665/sleep.2810, 23814343 PMC3669059

[16] Griffin SC Williams AB Ravyts SG Mladen SN Rybarczyk BD. Loneliness and sleep: a systematic review and meta-analysis. Health Psychol Open. (2020) 7:2055102920913235. doi: 10.1177/2055102920913235, 32284871 PMC7139193

[17] Ramsawh HJ Stein MB Belik SL Jacobi F Sareen J. Relationship of anxiety disorders, sleep quality, and functional impairment in a community sample. J Psychiatr Res. (2009) 43:926–33. doi: 10.1016/j.jpsychires.2009.01.009, 19269650

[18] Peng A Ji S Lai W Hu D Wang M Zhao X . The bidirectional relationship between sleep disturbance and anxiety: sleep disturbance is a stronger predictor of anxiety. Sleep Med. (2024) 121:63–8. doi: 10.1016/j.sleep.2024.06.022, 38924831

[19] Alfano CA Mellman TA. "Sleep in anxiety disorders". In: Winkelman JW, Plante DT, editors. Foundations of Psychiatric Sleep Medicine. Cambridge: Cambridge University Press. (2010). p. 286–97. doi: 10.1017/CBO9780511777493.019

[20] Palmer CA Bower JL Cho KW Clementi MA Lau S Oosterhoff B . Sleep loss and emotion: a systematic review and meta-analysis of over 50 years of experimental research. Psychol Bull. (2024) 150:440–63. doi: 10.1037/bul0000410, 38127505

[21] Cacioppo JT Hawkley LC Crawford LE Ernst JM Burleson MH Kowalewski RB . Loneliness and health: potential mechanisms. Psychosom Med. (2002a) 64:407–17. doi: 10.1097/00006842-200205000-00005, 12021415

[22] Ben Simon E Walker MP. Sleep loss causes social withdrawal and loneliness. Nat Commun. (2018) 9:3146. doi: 10.1038/s41467-018-05377-0, 30108218 PMC6092357

[23] Borsboom D. Psychometric perspectives on diagnostic systems. J Clin Psychol. (2008) 64:1089–108. doi: 10.1002/jclp.20503 18683856

[24] Cramer AO Waldorp LJ Van Der Maas HL Borsboom D. Comorbidity: a network perspective. Behav Brain Sci. (2010a) 33:137–50. doi: 10.1017/S0140525X09991567, 20584369

[25] Borsboom D Cramer AO. Network analysis: an integrative approach to the structure of psychopathology. Annu Rev Clin Psychol. (2013) 9:91–121. doi: 10.1146/annurev-clinpsy-050212-185608, 23537483

[26] Beard C Millner AJ Forgeard MJ Fried EI Hsu KJ Treadway MT . Network analysis of depression and anxiety symptom relationships in a psychiatric sample. Psychol Med. (2016) 46:3359–69. doi: 10.1017/S0033291716002300, 27623748 PMC5430082

[27] Bian Z Xu R Shang B Lv F Sun W Li Q . Associations between anxiety, depression, and personal mastery in community-dwelling older adults: a network-based analysis. BMC Psychiatry. (2024) 24:192. doi: 10.1186/s12888-024-05644-z, 38454373 PMC10921593

[28] Huang J Tang A Li Q Chen F Ye S Song L . Network analysis of sleep disorders, depression and anxiety symptoms among community older adults. BMC Geriatr. (2025) 25:694. doi: 10.1186/s12877-025-06381-y, 40993514 PMC12462105

[29] Briganti G Scutari M McNally RJ. A tutorial on bayesian networks for psychopathology researchers. Psychol Methods. (2023) 28:947–61. doi: 10.1037/met0000479, 35113632

[30] McNally RJ Mair P Mugno BL Riemann BC. Co-morbid obsessive–compulsive disorder and depression: a Bayesian network approach. Psychol Med. (2017) 47:1204–14. doi: 10.1017/S0033291716003287, 28052778

[31] Buysse DJ Reynolds CF III Monk TH Berman SR Kupfer DJ. The Pittsburgh sleep quality index: a new instrument for psychiatric practice and research. Psychiatry Res. (1989) 28:193–213. doi: 10.1016/0165-1781(89)90047-4, 2748771

[32] Zhang C Zhang H Zhao M Li Z Cook CE Buysse DJ . Reliability, validity, and factor structure of Pittsburgh sleep quality index in community-based centenarians. Front Psych. (2020) 11:573530. doi: 10.3389/fpsyt.2020.573530, 33110414 PMC7488982

[33] Spitzer RL Kroenke K Williams JB Löwe B. A brief measure for assessing generalized anxiety disorder: the GAD-7. Arch Intern Med. (2006) 166:1092–7. doi: 10.1001/archinte.166.10.1092, 16717171

[34] Yang T Guo Z Cao X Zhu X Zhou Q Li X . Network analysis of anxiety and depression in the functionally impaired elderly. Front Public Health. (2022) 10:1067646. doi: 10.3389/fpubh.2022.1067646, 36530716 PMC9751796

[35] Russell D Peplau LA Ferguson ML. Developing a measure of loneliness. J Pers Assess. (1978) 42:290–4. doi: 10.1207/s15327752jpa4203_11, 660402

[36] Hays RD DiMatteo MR. A short-form measure of loneliness. J Pers Assess. (1987) 51:69–81. doi: 10.1207/s15327752jpa5101_6, 3572711

[37] Zhou L Li Z Hu M Xiao S. Reliability and validity of ULS-8 loneliness scale in elderly samples in a rural community. Zhong nan da xue xue bao Yi xue ban= Journal of Central South University. (2012) 37:1124–8. doi: 10.3969/j.issn.1672-7347.2012.11.008 23202622

[38] Epskamp S Cramer AO Waldorp LJ Schmittmann VD Borsboom D. Qgraph: network visualizations of relationships in psychometric data. J Stat Softw. (2012) 48:1–18. doi: 10.18637/jss.v048.i04

[39] Foygel R Drton M. Extended Bayesian information criteria for Gaussian graphical models. Adv Neural Inf Proces Syst. (2010) 23:604–12. doi: 10.48550/arXiv.1011.6640

[40] Haslbeck JM Waldorp LJ. Mgm: estimating time-varying mixed graphical models in high-dimensional data. J Stat Softw. (2020) 93:1–46. doi: 10.18637/jss.v093.i08

[41] Epskamp S Borsboom D Fried EI. Estimating psychological networks and their accuracy: a tutorial paper. Behav Res Methods. (2018) 50:195–212. doi: 10.3758/s13428-017-0862-1, 28342071 PMC5809547

[42] Forbes MK Wright AGC Markon KE Krueger RF. Evidence that psychopathology symptom networks have limited replicability. J Abnorm Psychol. (2017) 126:969–88. doi: 10.1037/abn0000276, 29106281 PMC5749927

[43] Robinaugh DJ Millner AJ McNally RJ. Identifying highly influential nodes in the complicated grief network. J Abnorm Psychol. (2016) 125:747. doi: 10.1037/abn0000181, 27505622 PMC5060093

[44] Jones PJ Ma R McNally RJ. Bridge centrality: a network approach to understanding comorbidity. Multivar Behav Res. (2019) 56:353–67. doi: 10.1080/00273171.2019.1614898, 31179765

[45] Cramer AO Waldorp LJ Van Der Maas HL Borsboom D. Complex realities require complex theories: refining and extending the network approach to mental disorders. Behav Brain Sci. (2010b) 33:178. doi: 10.1017/S0140525X10000920

[46] Scutari M. Learning Bayesian networks with the bnlearn R package. J Stat Softw. (2010) 35:1–22. doi: 10.18637/jss.v035.i0321603108

[47] Van Borkulo CD van Bork R Boschloo L Kossakowski JJ Tio P Schoevers RA . Comparing network structures on three aspects: a permutation test. Psychol Methods. (2022) 28:1273. doi: 10.1037/met0000476, 35404628

[48] Reichardt J Bornholdt S. Statistical mechanics of community detection. Phys Rev E. (2006) 74:016110. doi: 10.1103/PhysRevE.74.016110, 16907154

[49] Tao Y Liu Q Ye X Feng J Liu H Wu J . Uncovering the symptom relationship among sleep quality, anxiety, and depression in Chinese patients with breast cancer: multidimensional data validation using PSQI versus actigraphy. J Cancer Surviv. (2024) 20:–235. doi: 10.1007/s11764-024-01649-5, 39141310

[50] Liu Y Zhu Z Yan J Wang Y. The relationship between anxiety and depression symptoms in insomnia patients: a network analysis. Sci Rep. (2025) 15:25030. doi: 10.1038/s41598-025-09746-w, 40646108 PMC12254401

[51] Jiang X Wang X Liu B Yu L He J Wu S . Associations of depression, anxiety, and insomnia symptoms in subthreshold depression: a network analysis. BMC Psychiatry. (2025) 25:970–10. doi: 10.1186/s12888-025-07437-4, 41074002 PMC12512687

[52] Lin R Wei B Huang C Chen D Zhu Z Shi Y . Emergent neuropsychiatric symptoms and sleep disorders among older adults in nursing homes who have depressive symptoms and are at risk of dementia: a network analysis. J Affect Disord. (2025) 390:119825. doi: 10.1016/j.jad.2025.119825, 40623647

[53] Cacioppo JT Hawkley LC Berntson GG Ernst JM Gibbs AC Stickgold R . Do lonely days invade the nights? Potential social modulation of sleep efficiency. Psychol Sci. (2002b) 13:384–7. doi: 10.1111/1467-9280.00469, 12137144

[54] Massar SA Liu JC Mohammad NB Chee MW. Poor habitual sleep efficiency is associated with increased cardiovascular and cortisol stress reactivity in men. Psychoneuroendocrinology. (2017) 81:151–6. doi: 10.1016/j.psyneuen.2017.04.013, 28482312

[55] Papadimitriou GN Linkowski P. Sleep disturbance in anxiety disorders. Int Rev Psychiatry. (2005) 17:229–36. doi: 10.1080/09540260500104524, 16194794

[56] Jones PJ Mair P Riemann BC Mugno BL McNally RJ. A network perspective on comorbid depression in adolescents with obsessive-compulsive disorder. J Anxiety Disord. (2018) 53:1–8. doi: 10.1016/j.janxdis.2017.09.008, 29125957

[57] Wilckens KA Habte RF Dong Y Stepan ME Dessa KM Whitehead AB . A pilot time-in-bed restriction intervention behaviorally enhances slow-wave activity in older adults. Front Sleep. (2024) 2:1265006. doi: 10.3389/frsle.2023.1265006, 38938690 PMC11210605

[58] Pchelina P Poluektov M Krieger T Duss SB Berger T. Clinical effectiveness of internet-based cognitive behavioral therapy for insomnia in routine secondary care: results of a randomized controlled trial. Front Psych. (2024) 15:1301489. doi: 10.3389/fpsyt.2024.1301489, 38800061 PMC11116772

[59] Ekström H Svensson M Elmståhl S Wranker LS. The association between loneliness, social isolation, and sleep disturbances in older adults: a follow-up study from the Swedish good aging in Skåne project. SAGE Open Med. (2024) 12:20503121231222823. doi: 10.1177/20503121231222823, 38249948 PMC10798090

[60] Pearl J. Causality: Models, Reasoning, and Inference. Cambridge: Cambridge University Press (2000).

[61] Bringmann LF Elmer T Epskamp S Krause RW Schoch D Wichers M . What do centrality measures measure in psychological networks? J Abnorm Psychol. (2019) 128:892. doi: 10.1037/abn0000446 31318245

[62] Zhang L Tao Y Hou W Niu H Ma Z Zheng Z . Seeking bridge symptoms of anxiety, depression, and sleep disturbance among the elderly during the lockdown of the COVID-19 pandemic—a network approach. Front Psych. (2022) 13:919251. doi: 10.3389/fpsyt.2022.919251, 35990065 PMC9381922

