# Supplementary material for: Network analysis of sleep disorders, anxiety, and loneliness among the community-dwelling older adults
Source: Front Public Health. 2026 Jun 10;14:1827779. doi: 10.3389/fpubh.2026.1827779 (PMC13290629; doi:10.3389/fpubh.2026.1827779)
Supplement: Supplementary file 1 [file Table_1.docx]

Supplementary Material

**Table S1 The Results of the Case-Dropping Bootstrap Analysis**

| **Subset** | **Sample Size (nPerson)** | **Percentage Dropped (Drop%)** | **of Subsets (n)** |
| --- | --- | --- | --- |
| 1 | 409 | 75.0% | 158 |
| 2 | 537 | 67.2% | 153 |
| 3 | 664 | 59.4% | 156 |
| 4 | 791 | 51.7% | 147 |
| 5 | 919 | 43.9% | 165 |
| 6 | 1046 | 36.1% | 153 |
| 7 | 1173 | 28.3% | 147 |
| 8 | 1301 | 20.5% | 143 |
| 9 | 1428 | 12.8% | 127 |
| 10 | 1555 | 5.0% | 151 |

***Note:*** *This table summarizes the stability of network centrality indices using the case-dropping subset bootstrap procedure. For each subsample level, the table reports the number of remaining persons (nPerson), the corresponding percentage of the full sample dropped (Drop%), and the number of bootstrap samples drawn (n).*

**Table S2 The Results of DAG Based on Bayesian Information Criterion (BIC)**

| NO | From | To | Strength |
| --- | --- | --- | --- |
| 1 | GAD2 | GAD1 | -25.89 |
| 2 | GAD2 | GAD4 | -3.29 |
| 3 | GAD2 | GAD7 | -12.31 |
| 4 | GAD2 | PSQI7 | 2.37 |
| 5 | GAD2 | ULS1 | -2.18 |
| 6 | GAD3 | GAD1 | -8.15 |
| 7 | GAD3 | GAD2 | -106.06 |
| 8 | GAD3 | GAD6 | -93.00 |
| 9 | GAD3 | PSQI1 | -28.90 |
| 10 | GAD3 | PSQI4 | -56.40 |
| 11 | GAD3 | ULS1 | -6.61 |
| 12 | GAD3 | ULS2 | -11.47 |
| 13 | GAD3 | ULS5 | -75.41 |
| 14 | GAD4 | GAD1 | -65.26 |
| 15 | GAD5 | GAD4 | -1.05 |
| 16 | GAD5 | GAD6 | -34.55 |
| 17 | GAD5 | GAD7 | -3.71 |
| 18 | GAD5 | PSQI2 | -0.71 |
| 19 | GAD5 | ULS2 | -9.10 |
| 20 | GAD6 | GAD1 | -2.52 |
| 21 | GAD6 | GAD4 | -7.01 |
| 22 | GAD6 | GAD7 | -32.96 |
| 23 | GAD7 | GAD4 | -34.64 |
| 24 | PSQI1 | GAD1 | -21.14 |
| 25 | PSQI1 | PSQI2 | -62.72 |
| 26 | PSQI1 | PSQI3 | -8.78 |
| 27 | PSQI1 | PSQI4 | -49.79 |
| 28 | PSQI1 | PSQI5 | -14.48 |
| 29 | PSQI1 | PSQI7 | -16.78 |
| 30 | PSQI1 | ULS4 | -5.45 |
| 31 | PSQI1 | ULS7 | 2.42 |
| 32 | PSQI2 | GAD1 | -35.48 |
| 33 | PSQI2 | GAD7 | -4.38 |
| 34 | PSQI2 | ULS2 | -11.32 |
| 35 | PSQI2 | ULS4 | -4.91 |
| 36 | PSQI3 | GAD7 | -8.30 |
| 37 | PSQI3 | PSQI2 | -30.53 |
| 38 | PSQI3 | PSQI5 | -16.32 |
| 39 | PSQI3 | PSQI7 | -16.65 |
| 40 | PSQI3 | ULS1 | -24.58 |
| 41 | PSQI3 | ULS5 | -2.89 |
| 42 | PSQI4 | GAD1 | -13.34 |
| 43 | PSQI4 | GAD5 | -61.97 |
| 44 | PSQI4 | GAD6 | -0.95 |
| 45 | PSQI4 | GAD7 | -4.17 |
| 46 | PSQI4 | PSQI3 | -39.83 |
| 47 | PSQI4 | ULS1 | -30.31 |
| 48 | PSQI4 | ULS2 | -11.04 |
| 49 | PSQI4 | ULS6 | -41.22 |
| 50 | PSQI4 | ULS7 | -10.60 |
| 51 | PSQI5 | GAD1 | -1.30 |
| 52 | PSQI5 | GAD7 | 1.74 |
| 53 | PSQI5 | PSQI7 | -51.45 |
| 54 | PSQI5 | ULS1 | -10.93 |
| 55 | PSQI6 | GAD2 | -10.54 |
| 56 | PSQI6 | GAD4 | -1.18 |
| 57 | PSQI6 | PSQI1 | -68.87 |
| 58 | PSQI6 | PSQI2 | -3.04 |
| 59 | PSQI6 | PSQI3 | -45.41 |
| 60 | PSQI6 | PSQI5 | -12.04 |
| 61 | PSQI6 | PSQI7 | -11.85 |
| 62 | PSQI6 | ULS4 | -5.18 |
| 63 | PSQI6 | ULS5 | -11.78 |
| 64 | PSQI7 | GAD4 | -12.94 |
| 65 | PSQI7 | ULS1 | -21.67 |
| 66 | PSQI7 | ULS6 | -5.63 |
| 67 | ULS2 | PSQI5 | -4.76 |
| 68 | ULS3 | GAD1 | -20.52 |
| 69 | ULS3 | GAD7 | -0.86 |
| 70 | ULS3 | PSQI1 | -8.24 |
| 71 | ULS3 | PSQI5 | 1.34 |
| 72 | ULS3 | ULS2 | -4.45 |
| 73 | ULS3 | ULS4 | -52.44 |
| 74 | ULS3 | ULS5 | -33.21 |
| 75 | ULS3 | ULS6 | -35.42 |
| 76 | ULS3 | ULS7 | -52.69 |
| 77 | ULS3 | ULS8 | -130.99 |
| 78 | ULS4 | GAD1 | -9.93 |
| 79 | ULS4 | GAD2 | -16.76 |
| 80 | ULS4 | GAD4 | -5.72 |
| 81 | ULS4 | GAD7 | -3.34 |
| 82 | ULS4 | ULS1 | -24.31 |
| 83 | ULS4 | ULS2 | -1.31 |
| 84 | ULS5 | GAD4 | -1.44 |
| 85 | ULS5 | GAD5 | -83.06 |
| 86 | ULS5 | GAD7 | -27.66 |
| 87 | ULS5 | PSQI2 | -3.89 |
| 88 | ULS5 | PSQI7 | -1.29 |
| 89 | ULS5 | ULS1 | -21.57 |
| 90 | ULS5 | ULS2 | -18.73 |
| 91 | ULS6 | GAD1 | -5.81 |
| 92 | ULS6 | GAD7 | -5.92 |
| 93 | ULS6 | ULS1 | -0.75 |
| 94 | ULS7 | GAD1 | -40.32 |
| 95 | ULS7 | GAD4 | -8.59 |
| 96 | ULS7 | GAD7 | -4.60 |
| 97 | ULS7 | PSQI2 | -20.49 |
| 98 | ULS7 | ULS1 | -21.83 |
| 99 | ULS7 | ULS4 | -17.00 |
| 100 | ULS7 | ULS6 | -20.22 |
| 101 | ULS8 | GAD2 | -38.74 |
| 102 | ULS8 | GAD6 | -12.87 |
| 103 | ULS8 | GAD7 | -15.82 |
| 104 | ULS8 | PSQI1 | -15.40 |
| 105 | ULS8 | PSQI2 | -5.26 |
| 106 | ULS8 | PSQI3 | -11.51 |
| 107 | ULS8 | PSQI6 | -2.96 |
| 108 | ULS8 | PSQI7 | -11.84 |
| 109 | ULS8 | ULS2 | -22.38 |
| 110 | ULS8 | ULS5 | -43.33 |
| 111 | ULS8 | ULS6 | -15.62 |
| 112 | ULS8 | ULS7 | -46.79 |

**Table S3 The Results of DAG Based on Probability**

| **NO** | **From** | **To** | **Strength** | **Direction** |
| --- | --- | --- | --- | --- |
| 13 | GAD1 | PSQI7 | 0.52 | 0.52 |
| 22 | GAD2 | GAD1 | 0.69 | 0.69 |
| 24 | GAD2 | GAD4 | 0.61 | 0.61 |
| 25 | GAD2 | GAD5 | 0.72 | 0.72 |
| 27 | GAD2 | GAD7 | 0.59 | 0.59 |
| 34 | GAD2 | PSQI7 | 0.58 | 0.58 |
| 35 | GAD2 | ULS1 | 0.71 | 0.71 |
| 43 | GAD3 | GAD1 | 0.80 | 0.80 |
| 44 | GAD3 | GAD2 | 0.82 | 0.82 |
| 47 | GAD3 | GAD6 | 0.70 | 0.70 |
| 49 | GAD3 | PSQI1 | 0.59 | 0.59 |
| 52 | GAD3 | PSQI4 | 0.55 | 0.55 |
| 56 | GAD3 | ULS1 | 0.79 | 0.79 |
| 57 | GAD3 | ULS2 | 0.78 | 0.78 |
| 60 | GAD3 | ULS5 | 0.73 | 0.73 |
| 64 | GAD4 | GAD1 | 0.62 | 0.62 |
| 71 | GAD4 | PSQI2 | 0.53 | 0.53 |
| 88 | GAD5 | GAD4 | 0.59 | 0.59 |
| 89 | GAD5 | GAD6 | 0.53 | 0.53 |
| 90 | GAD5 | GAD7 | 0.53 | 0.53 |
| 92 | GAD5 | PSQI2 | 0.57 | 0.57 |
| 99 | GAD5 | ULS2 | 0.51 | 0.51 |
| 106 | GAD6 | GAD1 | 0.69 | 0.69 |
| 109 | GAD6 | GAD4 | 0.59 | 0.59 |
| 111 | GAD6 | GAD7 | 0.53 | 0.53 |
| 130 | GAD7 | GAD4 | 0.59 | 0.59 |
| 139 | GAD7 | PSQI7 | 0.54 | 0.54 |
| 148 | PSQI1 | GAD1 | 0.79 | 0.79 |
| 155 | PSQI1 | PSQI2 | 0.68 | 0.68 |
| 156 | PSQI1 | PSQI3 | 0.60 | 0.60 |
| 157 | PSQI1 | PSQI4 | 0.53 | 0.53 |
| 158 | PSQI1 | PSQI5 | 0.66 | 0.66 |
| 160 | PSQI1 | PSQI7 | 0.68 | 0.68 |
| 164 | PSQI1 | ULS4 | 0.55 | 0.55 |
| 167 | PSQI1 | ULS7 | 0.62 | 0.62 |
| 169 | PSQI2 | GAD1 | 0.54 | 0.54 |
| 175 | PSQI2 | GAD7 | 0.57 | 0.57 |
| 183 | PSQI2 | ULS2 | 0.71 | 0.71 |
| 185 | PSQI2 | ULS4 | 0.60 | 0.60 |
| 196 | PSQI3 | GAD7 | 0.63 | 0.63 |
| 198 | PSQI3 | PSQI2 | 0.60 | 0.60 |
| 200 | PSQI3 | PSQI5 | 0.58 | 0.58 |
| 202 | PSQI3 | PSQI7 | 0.60 | 0.60 |
| 203 | PSQI3 | ULS1 | 0.66 | 0.66 |
| 207 | PSQI3 | ULS5 | 0.66 | 0.66 |
| 211 | PSQI4 | GAD1 | 0.67 | 0.67 |
| 215 | PSQI4 | GAD5 | 0.71 | 0.71 |
| 216 | PSQI4 | GAD6 | 0.56 | 0.56 |
| 217 | PSQI4 | GAD7 | 0.67 | 0.67 |
| 220 | PSQI4 | PSQI3 | 0.54 | 0.54 |
| 222 | PSQI4 | PSQI6 | 0.55 | 0.55 |
| 224 | PSQI4 | ULS1 | 0.74 | 0.74 |
| 225 | PSQI4 | ULS2 | 0.73 | 0.73 |
| 229 | PSQI4 | ULS6 | 0.71 | 0.71 |
| 230 | PSQI4 | ULS7 | 0.52 | 0.52 |
| 232 | PSQI5 | GAD1 | 0.81 | 0.81 |
| 238 | PSQI5 | GAD7 | 0.72 | 0.72 |
| 244 | PSQI5 | PSQI7 | 0.65 | 0.65 |
| 245 | PSQI5 | ULS1 | 0.67 | 0.67 |
| 254 | PSQI6 | GAD2 | 0.66 | 0.66 |
| 256 | PSQI6 | GAD4 | 0.69 | 0.69 |
| 260 | PSQI6 | PSQI1 | 0.56 | 0.56 |
| 261 | PSQI6 | PSQI2 | 0.64 | 0.64 |
| 262 | PSQI6 | PSQI3 | 0.58 | 0.58 |
| 264 | PSQI6 | PSQI5 | 0.71 | 0.71 |
| 265 | PSQI6 | PSQI7 | 0.67 | 0.67 |
| 269 | PSQI6 | ULS4 | 0.59 | 0.59 |
| 270 | PSQI6 | ULS5 | 0.59 | 0.59 |
| 277 | PSQI7 | GAD4 | 0.61 | 0.61 |
| 287 | PSQI7 | ULS1 | 0.61 | 0.61 |
| 292 | PSQI7 | ULS6 | 0.53 | 0.53 |
| 327 | ULS2 | PSQI5 | 0.50 | 0.50 |
| 337 | ULS3 | GAD1 | 0.82 | 0.82 |
| 343 | ULS3 | GAD7 | 0.68 | 0.68 |
| 344 | ULS3 | PSQI1 | 0.56 | 0.56 |
| 348 | ULS3 | PSQI5 | 0.66 | 0.66 |
| 352 | ULS3 | ULS2 | 0.81 | 0.81 |
| 353 | ULS3 | ULS4 | 0.71 | 0.71 |
| 354 | ULS3 | ULS5 | 0.72 | 0.72 |
| 355 | ULS3 | ULS6 | 0.80 | 0.80 |
| 356 | ULS3 | ULS7 | 0.70 | 0.70 |
| 357 | ULS3 | ULS8 | 0.51 | 0.51 |
| 358 | ULS4 | GAD1 | 0.64 | 0.64 |
| 359 | ULS4 | GAD2 | 0.71 | 0.71 |
| 361 | ULS4 | GAD4 | 0.61 | 0.61 |
| 364 | ULS4 | GAD7 | 0.50 | 0.50 |
| 372 | ULS4 | ULS1 | 0.75 | 0.75 |
| 373 | ULS4 | ULS2 | 0.69 | 0.69 |
| 382 | ULS5 | GAD4 | 0.59 | 0.59 |
| 383 | ULS5 | GAD5 | 0.71 | 0.71 |
| 385 | ULS5 | GAD7 | 0.59 | 0.59 |
| 387 | ULS5 | PSQI2 | 0.51 | 0.51 |
| 392 | ULS5 | PSQI7 | 0.54 | 0.54 |
| 393 | ULS5 | ULS1 | 0.73 | 0.73 |
| 394 | ULS5 | ULS2 | 0.60 | 0.60 |
| 400 | ULS6 | GAD1 | 0.58 | 0.58 |
| 401 | ULS6 | GAD2 | 0.59 | 0.59 |
| 406 | ULS6 | GAD7 | 0.62 | 0.62 |
| 414 | ULS6 | ULS1 | 0.68 | 0.68 |
| 417 | ULS6 | ULS4 | 0.54 | 0.54 |
| 421 | ULS7 | GAD1 | 0.65 | 0.65 |
| 424 | ULS7 | GAD4 | 0.52 | 0.52 |
| 427 | ULS7 | GAD7 | 0.56 | 0.56 |
| 429 | ULS7 | PSQI2 | 0.53 | 0.53 |
| 435 | ULS7 | ULS1 | 0.79 | 0.79 |
| 438 | ULS7 | ULS4 | 0.53 | 0.53 |
| 440 | ULS7 | ULS6 | 0.51 | 0.51 |
| 443 | ULS8 | GAD2 | 0.83 | 0.83 |
| 447 | ULS8 | GAD6 | 0.68 | 0.68 |
| 448 | ULS8 | GAD7 | 0.79 | 0.79 |
| 449 | ULS8 | PSQI1 | 0.65 | 0.65 |
| 450 | ULS8 | PSQI2 | 0.52 | 0.52 |
| 451 | ULS8 | PSQI3 | 0.65 | 0.65 |
| 454 | ULS8 | PSQI6 | 0.59 | 0.59 |
| 455 | ULS8 | PSQI7 | 0.66 | 0.66 |
| 457 | ULS8 | ULS2 | 0.83 | 0.83 |
| 460 | ULS8 | ULS5 | 0.77 | 0.77 |
| 461 | ULS8 | ULS6 | 0.74 | 0.74 |
| 462 | ULS8 | ULS7 | 0.64 | 0.64 |

**Table S4. Results of Centrality Invariance Tests for Expected Influence**

| Node | Test statistics (C) | p-value |
| --- | --- | --- |
| GAD1 | -0.09 | 0.35 |
| GAD2 | -0.01 | 0.91 |
| GAD3 | 0.02 | 0.85 |
| GAD4 | -0.02 | 0.82 |
| GAD5 | -0.04 | 0.67 |
| GAD6 | -0.04 | 0.64 |
| GAD7 | 0.08 | 0.36 |
| PSQI1 | -0.03 | 0.73 |
| PSQI2 | -0.01 | 0.93 |
| PSQI3 | -0.06 | 0.49 |
| PSQI4 | -0.06 | 0.50 |
| PSQI5 | 0.07 | 0.41 |
| PSQI6 | 0.07 | 0.41 |
| PSQI7 | 0.05 | 0.58 |
| ULS1 | -0.04 | 0.64 |
| ULS2 | -0.10 | 0.34 |
| ULS3 | 0.02 | 0.87 |
| ULS4 | -0.02 | 0.88 |
| ULS5 | -0.05 | 0.58 |
| ULS6 | 0.13 | 0.14 |
| ULS7 | 0.06 | 0.50 |
| ULS8 | 0.00 | 0.99 |

***Note:*** *This table presents the results of invariance tests for the Expected Influence centrality measure for each node across groups.*

**Table S5: Results of Edge Weight Invariance Tests**

| **NO** | **Node1** | **Node2** | **p-value** | **Test statistic E** |
| --- | --- | --- | --- | --- |
| 1 | GAD1 | GAD2 | 0.35 | 0.04 |
| 2 | GAD1 | GAD3 | 0.56 | 0.03 |
| 3 | GAD2 | GAD3 | 0.19 | 0.06 |
| 4 | GAD1 | GAD4 | 0.66 | 0.02 |
| 5 | GAD2 | GAD4 | 0.97 | 0.00 |
| 6 | GAD3 | GAD4 | 0.62 | 0.02 |
| 7 | GAD1 | GAD5 | 0.41 | 0.04 |
| 8 | GAD2 | GAD5 | 0.61 | 0.02 |
| 9 | GAD3 | GAD5 | 1.00 | 0.00 |
| 10 | GAD4 | GAD5 | 0.94 | 0.00 |
| 11 | GAD1 | GAD6 | 0.66 | 0.02 |
| 12 | GAD2 | GAD6 | 0.26 | 0.05 |
| 13 | GAD3 | GAD6 | 0.53 | 0.03 |
| 14 | GAD4 | GAD6 | 0.16 | 0.06 |
| 15 | GAD5 | GAD6 | 0.15 | 0.06 |
| 16 | GAD1 | GAD7 | 0.35 | 0.04 |
| 17 | GAD2 | GAD7 | 0.41 | 0.04 |
| 18 | GAD3 | GAD7 | 0.45 | 0.03 |
| 19 | GAD4 | GAD7 | 0.14 | 0.07 |
| 20 | GAD5 | GAD7 | 0.35 | 0.04 |
| 21 | GAD6 | GAD7 | 0.41 | 0.04 |
| 22 | GAD1 | PSQI1 | 1.00 | 0.00 |
| 23 | GAD2 | PSQI1 | 0.51 | 0.03 |
| 24 | GAD3 | PSQI1 | 0.84 | 0.01 |
| 25 | GAD4 | PSQI1 | 1.00 | 0.00 |
| 26 | GAD5 | PSQI1 | 1.00 | 0.00 |
| 27 | GAD6 | PSQI1 | 0.59 | 0.00 |
| 28 | GAD7 | PSQI1 | 1.00 | 0.00 |
| 29 | GAD1 | PSQI2 | 0.73 | 0.01 |
| 30 | GAD2 | PSQI2 | 0.77 | 0.00 |
| 31 | GAD3 | PSQI2 | 1.00 | 0.00 |
| 32 | GAD4 | PSQI2 | 1.00 | 0.00 |
| 33 | GAD5 | PSQI2 | 0.51 | 0.03 |
| 34 | GAD6 | PSQI2 | 1.00 | 0.00 |
| 35 | GAD7 | PSQI2 | 1.00 | 0.00 |
| 36 | PSQI1 | PSQI2 | 0.90 | 0.01 |
| 37 | GAD1 | PSQI3 | 1.00 | 0.00 |
| 38 | GAD2 | PSQI3 | 1.00 | 0.00 |
| 39 | GAD3 | PSQI3 | 1.00 | 0.00 |
| 40 | GAD4 | PSQI3 | 1.00 | 0.00 |
| 41 | GAD5 | PSQI3 | 1.00 | 0.00 |
| 42 | GAD6 | PSQI3 | 1.00 | 0.00 |
| 43 | GAD7 | PSQI3 | 1.00 | 0.00 |
| 44 | PSQI1 | PSQI3 | 0.59 | 0.02 |
| 45 | PSQI2 | PSQI3 | 0.53 | 0.03 |
| 46 | GAD1 | PSQI4 | 0.25 | 0.05 |
| 47 | GAD2 | PSQI4 | 1.00 | 0.00 |
| 48 | GAD3 | PSQI4 | 0.85 | 0.01 |
| 49 | GAD4 | PSQI4 | 0.05 | 0.05 |
| 50 | GAD5 | PSQI4 | 0.99 | 0.00 |
| 51 | GAD6 | PSQI4 | 0.22 | 0.05 |
| 52 | GAD7 | PSQI4 | 0.95 | 0.00 |
| 53 | PSQI1 | PSQI4 | 0.25 | 0.06 |
| 54 | PSQI2 | PSQI4 | 0.22 | 0.05 |
| 55 | PSQI3 | PSQI4 | 0.79 | 0.01 |
| 56 | GAD1 | PSQI5 | 1.00 | 0.00 |
| 57 | GAD2 | PSQI5 | 0.55 | 0.01 |
| 58 | GAD3 | PSQI5 | 0.02 | 0.05 |
| 59 | GAD4 | PSQI5 | 1.00 | 0.00 |
| 60 | GAD5 | PSQI5 | 1.00 | 0.00 |
| 61 | GAD6 | PSQI5 | 1.00 | 0.00 |
| 62 | GAD7 | PSQI5 | 1.00 | 0.00 |
| 63 | PSQI1 | PSQI5 | 0.10 | 0.08 |
| 64 | PSQI2 | PSQI5 | 0.34 | 0.03 |
| 65 | PSQI3 | PSQI5 | 0.50 | 0.03 |
| 66 | PSQI4 | PSQI5 | 0.59 | 0.03 |
| 67 | GAD1 | PSQI6 | 1.00 | 0.00 |
| 68 | GAD2 | PSQI6 | 0.68 | 0.02 |
| 69 | GAD3 | PSQI6 | 1.00 | 0.00 |
| 70 | GAD4 | PSQI6 | 0.40 | 0.04 |
| 71 | GAD5 | PSQI6 | 1.00 | 0.00 |
| 72 | GAD6 | PSQI6 | 1.00 | 0.00 |
| 73 | GAD7 | PSQI6 | 1.00 | 0.00 |
| 74 | PSQI1 | PSQI6 | 0.56 | 0.03 |
| 75 | PSQI2 | PSQI6 | 0.98 | 0.00 |
| 76 | PSQI3 | PSQI6 | 0.07 | 0.09 |
| 77 | PSQI4 | PSQI6 | 0.30 | 0.05 |
| 78 | PSQI5 | PSQI6 | 0.68 | 0.02 |
| 79 | GAD1 | PSQI7 | 0.71 | 0.02 |
| 80 | GAD2 | PSQI7 | 1.00 | 0.00 |
| 81 | GAD3 | PSQI7 | 1.00 | 0.00 |
| 82 | GAD4 | PSQI7 | 0.11 | 0.07 |
| 83 | GAD5 | PSQI7 | 1.00 | 0.00 |
| 84 | GAD6 | PSQI7 | 0.88 | 0.01 |
| 85 | GAD7 | PSQI7 | 0.80 | 0.01 |
| 86 | PSQI1 | PSQI7 | 0.75 | 0.01 |
| 87 | PSQI2 | PSQI7 | 0.23 | 0.05 |
| 88 | PSQI3 | PSQI7 | 0.03 | 0.09 |
| 89 | PSQI4 | PSQI7 | 0.38 | 0.03 |
| 90 | PSQI5 | PSQI7 | 0.95 | 0.00 |
| 91 | PSQI6 | PSQI7 | 0.59 | 0.02 |
| 92 | GAD1 | ULS1 | 0.26 | 0.02 |
| 93 | GAD2 | ULS1 | 0.17 | 0.06 |
| 94 | GAD3 | ULS1 | 0.50 | 0.03 |
| 95 | GAD4 | ULS1 | 0.08 | 0.06 |
| 96 | GAD5 | ULS1 | 0.03 | 0.01 |
| 97 | GAD6 | ULS1 | 1.00 | 0.00 |
| 98 | GAD7 | ULS1 | 0.66 | 0.02 |
| 99 | PSQI1 | ULS1 | 1.00 | 0.00 |
| 100 | PSQI2 | ULS1 | 0.25 | 0.03 |
| 101 | PSQI3 | ULS1 | 0.82 | 0.01 |
| 102 | PSQI4 | ULS1 | 0.22 | 0.07 |
| 103 | PSQI5 | ULS1 | 0.21 | 0.04 |
| 104 | PSQI6 | ULS1 | 1.00 | 0.00 |
| 105 | PSQI7 | ULS1 | 0.27 | 0.05 |
| 106 | GAD1 | ULS2 | 0.77 | 0.01 |
| 107 | GAD2 | ULS2 | 0.86 | 0.01 |
| 108 | GAD3 | ULS2 | 0.68 | 0.02 |
| 109 | GAD4 | ULS2 | 0.83 | 0.00 |
| 110 | GAD5 | ULS2 | 1.00 | 0.00 |
| 111 | GAD6 | ULS2 | 1.00 | 0.00 |
| 112 | GAD7 | ULS2 | 1.00 | 0.00 |
| 113 | PSQI1 | ULS2 | 0.15 | 0.06 |
| 114 | PSQI2 | ULS2 | 0.26 | 0.05 |
| 115 | PSQI3 | ULS2 | 0.07 | 0.05 |
| 116 | PSQI4 | ULS2 | 0.78 | 0.01 |
| 117 | PSQI5 | ULS2 | 0.31 | 0.05 |
| 118 | PSQI6 | ULS2 | 0.14 | 0.02 |
| 119 | PSQI7 | ULS2 | 1.00 | 0.00 |
| 120 | ULS1 | ULS2 | 0.12 | 0.07 |
| 121 | GAD1 | ULS3 | 0.92 | 0.00 |
| 122 | GAD2 | ULS3 | 0.11 | 0.04 |
| 123 | GAD3 | ULS3 | 1.00 | 0.00 |
| 124 | GAD4 | ULS3 | 1.00 | 0.00 |
| 125 | GAD5 | ULS3 | 0.37 | 0.00 |
| 126 | GAD6 | ULS3 | 1.00 | 0.00 |
| 127 | GAD7 | ULS3 | 1.00 | 0.00 |
| 128 | PSQI1 | ULS3 | 0.31 | 0.04 |
| 129 | PSQI2 | ULS3 | 1.00 | 0.00 |
| 130 | PSQI3 | ULS3 | 0.19 | 0.02 |
| 131 | PSQI4 | ULS3 | 1.00 | 0.00 |
| 132 | PSQI5 | ULS3 | 0.41 | 0.02 |
| 133 | PSQI6 | ULS3 | 1.00 | 0.00 |
| 134 | PSQI7 | ULS3 | 1.00 | 0.00 |
| 135 | ULS1 | ULS3 | 0.50 | 0.03 |
| 136 | ULS2 | ULS3 | 0.74 | 0.02 |
| 137 | GAD1 | ULS4 | 0.20 | 0.06 |
| 138 | GAD2 | ULS4 | 0.69 | 0.02 |
| 139 | GAD3 | ULS4 | 1.00 | 0.00 |
| 140 | GAD4 | ULS4 | 0.05 | 0.00 |
| 141 | GAD5 | ULS4 | 1.00 | 0.00 |
| 142 | GAD6 | ULS4 | 1.00 | 0.00 |
| 143 | GAD7 | ULS4 | 0.15 | 0.06 |
| 144 | PSQI1 | ULS4 | 0.75 | 0.02 |
| 145 | PSQI2 | ULS4 | 0.06 | 0.09 |
| 146 | PSQI3 | ULS4 | 1.00 | 0.00 |
| 147 | PSQI4 | ULS4 | 1.00 | 0.00 |
| 148 | PSQI5 | ULS4 | 1.00 | 0.00 |
| 149 | PSQI6 | ULS4 | 0.88 | 0.01 |
| 150 | PSQI7 | ULS4 | 0.18 | 0.04 |
| 151 | ULS1 | ULS4 | 0.39 | 0.04 |
| 152 | ULS2 | ULS4 | 0.03 | 0.08 |
| 153 | ULS3 | ULS4 | 0.97 | 0.00 |
| 154 | GAD1 | ULS5 | 1.00 | 0.00 |
| 155 | GAD2 | ULS5 | 1.00 | 0.00 |
| 156 | GAD3 | ULS5 | 0.93 | 0.00 |
| 157 | GAD4 | ULS5 | 0.24 | 0.05 |
| 158 | GAD5 | ULS5 | 0.55 | 0.03 |
| 159 | GAD6 | ULS5 | 0.47 | 0.03 |
| 160 | GAD7 | ULS5 | 0.18 | 0.06 |
| 161 | PSQI1 | ULS5 | 1.00 | 0.00 |
| 162 | PSQI2 | ULS5 | 0.81 | 0.01 |
| 163 | PSQI3 | ULS5 | 1.00 | 0.00 |
| 164 | PSQI4 | ULS5 | 1.00 | 0.00 |
| 165 | PSQI5 | ULS5 | 1.00 | 0.00 |
| 166 | PSQI6 | ULS5 | 0.82 | 0.01 |
| 167 | PSQI7 | ULS5 | 1.00 | 0.00 |
| 168 | ULS1 | ULS5 | 0.35 | 0.04 |
| 169 | ULS2 | ULS5 | 0.82 | 0.01 |
| 170 | ULS3 | ULS5 | 0.75 | 0.01 |
| 171 | ULS4 | ULS5 | 0.40 | 0.02 |
| 172 | GAD1 | ULS6 | 0.14 | 0.02 |
| 173 | GAD2 | ULS6 | 0.58 | 0.02 |
| 174 | GAD3 | ULS6 | 1.00 | 0.00 |
| 175 | GAD4 | ULS6 | 1.00 | 0.00 |
| 176 | GAD5 | ULS6 | 0.69 | 0.02 |
| 177 | GAD6 | ULS6 | 1.00 | 0.00 |
| 178 | GAD7 | ULS6 | 1.00 | 0.00 |
| 179 | PSQI1 | ULS6 | 0.64 | 0.02 |
| 180 | PSQI2 | ULS6 | 1.00 | 0.00 |
| 181 | PSQI3 | ULS6 | 1.00 | 0.00 |
| 182 | PSQI4 | ULS6 | 0.53 | 0.03 |
| 183 | PSQI5 | ULS6 | 0.30 | 0.01 |
| 184 | PSQI6 | ULS6 | 0.10 | 0.05 |
| 185 | PSQI7 | ULS6 | 0.20 | 0.05 |
| 186 | ULS1 | ULS6 | 0.08 | 0.07 |
| 187 | ULS2 | ULS6 | 0.04 | 0.08 |
| 188 | ULS3 | ULS6 | 0.93 | 0.00 |
| 189 | ULS4 | ULS6 | 0.28 | 0.05 |
| 190 | ULS5 | ULS6 | 0.06 | 0.04 |
| 191 | GAD1 | ULS7 | 0.29 | 0.06 |
| 192 | GAD2 | ULS7 | 0.79 | 0.01 |
| 193 | GAD3 | ULS7 | 1.00 | 0.00 |
| 194 | GAD4 | ULS7 | 0.12 | 0.07 |
| 195 | GAD5 | ULS7 | 1.00 | 0.00 |
| 196 | GAD6 | ULS7 | 1.00 | 0.00 |
| 197 | GAD7 | ULS7 | 0.08 | 0.05 |
| 198 | PSQI1 | ULS7 | 1.00 | 0.00 |
| 199 | PSQI2 | ULS7 | 0.16 | 0.06 |
| 200 | PSQI3 | ULS7 | 1.00 | 0.00 |
| 201 | PSQI4 | ULS7 | 1.00 | 0.00 |
| 202 | PSQI5 | ULS7 | 1.00 | 0.00 |
| 203 | PSQI6 | ULS7 | 1.00 | 0.00 |
| 204 | PSQI7 | ULS7 | 1.00 | 0.00 |
| 205 | ULS1 | ULS7 | 0.03 | 0.10 |
| 206 | ULS2 | ULS7 | 0.11 | 0.06 |
| 207 | ULS3 | ULS7 | 0.93 | 0.00 |
| 208 | ULS4 | ULS7 | 0.97 | 0.00 |
| 209 | ULS5 | ULS7 | 0.90 | 0.00 |
| 210 | ULS6 | ULS7 | 0.14 | 0.07 |
| 211 | GAD1 | ULS8 | 1.00 | 0.00 |
| 212 | GAD2 | ULS8 | 0.37 | 0.04 |
| 213 | GAD3 | ULS8 | 0.51 | 0.03 |
| 214 | GAD4 | ULS8 | 1.00 | 0.00 |
| 215 | GAD5 | ULS8 | 0.16 | 0.01 |
| 216 | GAD6 | ULS8 | 0.68 | 0.02 |
| 217 | GAD7 | ULS8 | 0.56 | 0.02 |
| 218 | PSQI1 | ULS8 | 0.49 | 0.03 |
| 219 | PSQI2 | ULS8 | 0.07 | 0.08 |
| 220 | PSQI3 | ULS8 | 0.05 | 0.03 |
| 221 | PSQI4 | ULS8 | 1.00 | 0.00 |
| 222 | PSQI5 | ULS8 | 1.00 | 0.00 |
| 223 | PSQI6 | ULS8 | 1.00 | 0.00 |
| 224 | PSQI7 | ULS8 | 0.31 | 0.04 |
| 225 | ULS1 | ULS8 | 0.91 | 0.00 |
| 226 | ULS2 | ULS8 | 0.71 | 0.02 |
| 227 | ULS3 | ULS8 | 0.69 | 0.02 |
| 228 | ULS4 | ULS8 | 0.07 | 0.05 |
| 229 | ULS5 | ULS8 | 0.89 | 0.01 |
| 230 | ULS6 | ULS8 | 0.86 | 0.01 |
| 231 | ULS7 | ULS8 | 0.39 | 0.04 |

*Note: This table presents the complete results of the invariance tests for all estimated edge weights between symptoms across the compared groups. The p-value for each edge indicates the statistical significance of its weight difference between groups.*
